# Modulation of recognition memory performance by light requires both melanopsin and classical photoreceptors

**DOI:** 10.1098/rspb.2016.2275

**Published:** 2016-12-28

**Authors:** Shu K. E. Tam, Sibah Hasan, Steven Hughes, Mark W. Hankins, Russell G. Foster, David M. Bannerman, Stuart N. Peirson

**Affiliations:** 1 Sleep and Circadian Neuroscience Institute (Nuffield Department of Clinical Neurosciences), Oxford Molecular Pathology Institute, Sir William Dunn School of Pathology, South Parks Road, Oxford OX1 3RE, UK; 2 Department of Experimental Psychology, Oxford University, Tinbergen Building, 9 South Parks Road, Oxford OX1 3UD, UK

**Keywords:** recognition performance, visual context, irradiance detection, rods and cones, melanopsin-expressing photosensitive retinal ganglion cells

## Abstract

Acute light exposure exerts various effects on physiology and behaviour. Although the effects of light on brain network activity in humans are well demonstrated, the effects of light on cognitive performance are inconclusive, with the size, as well as direction, of the effect depending on the nature of the task. Similarly, in nocturnal rodents, bright light can either facilitate or disrupt performance depending on the type of task employed. Crucially, it is unclear whether the effects of light on behavioural performance are mediated via the classical image-forming rods and cones or the melanopsin-expressing photosensitive retinal ganglion cells. Here, we investigate the modulatory effects of light on memory performance in mice using the spontaneous object recognition task. Importantly, we examine which photoreceptors are required to mediate the effects of light on memory performance. By using a cross-over design, we show that object recognition memory is disrupted when the test phase is conducted under a bright light (350 lux), regardless of the light level in the sample phase (10 or 350 lux), demonstrating that exposure to a bright light at the time of test, rather than at the time of encoding, impairs performance. Strikingly, the modulatory effect of light on memory performance is completely abolished in both melanopsin-deficient and rodless–coneless mice. Our findings provide direct evidence that melanopsin-driven and rod/cone-driven photoresponses are integrated in order to mediate the effect of light on memory performance.

## 1. Introduction

Acute exposure to light exerts effects on a range of physiological parameters, including body temperature, melatonin and cortisol synthesis, as well as sleep and waking electroencephalography [1–4]. These responses are accompanied by marked but transient effects in various brain regions involved in attentional and memory processes, such as the parietal cortex and hippocampus [5–8]. Although it is well demonstrated in human brain imaging studies that acute light exposure can activate cortical and subcortical networks involved in arousal, attention and memory [5–8], the effects of light on cognitive performance are less conclusive, with the size, as well as direction, of the effect depending on the nature of the task [9–11]. Similarly, in nocturnal rodents which show aversion to light [12], bright light can either facilitate or disrupt memory performance, depending on the type of task employed (e.g. fear conditioning versus water maze tasks; [13–15]). Crucially, it is unclear whether the modulatory effects of light on performance in these studies are mediated via the classical image-forming (IF) rods and cones, or the melanopsin (OPN4)-expressing photosensitive retinal ganglion cells (pRGCs), which mediate many other non-image-forming (NIF) responses, such as circadian entrainment, pupillary constriction, negative masking and modulation of sleep in response to light [16–20].

Indirect evidence of melanopsin involvement in modulating cortical and subcortical activities comes from a recent human study in which different wavelengths of light were used [8]. More direct evidence for melanopsin involvement comes from a mouse study [13], which reported that acute exposure to light enhanced the conditioned-freezing response to an auditory stimulus that had been paired with a mild electric shock. Importantly, this modulatory effect of light on conditioned-freezing performance was attenuated, although not completely abolished, in melanopsin-deficient (*Opn4*^−/–^) mice. However, the change in freezing in wild-type (WT) mice could be due to retrieval of a stronger associative memory (leading to better performance), but it could also be the result of a general increase in anxiety in response to light inputs [12]. This latter effect has been demonstrated in a recent study in which chemogenetic activation of OPN4-expressing pRGCs elevates the level of anxiety/arousal in the open field and elevated–plus maze tests [21]. These findings complicate interpretation of the role of the melanopsin in regulating memory performance. Moreover, it is unclear if acute light exposure would exert a similar facilitative effect in non-aversive memory tasks, as findings from previous studies suggest that the direction of the effect of light on behavioural performance may vary in different tasks [13–15].

Accordingly, we examine the modulatory effects of light on performance using the non-aversive, spontaneous object recognition task [22–25]. As the neural pathways that are involved in object recognition memory are likely to differ, at least in part, from those involved in fear memory [26–28], bright light may exert a different effect in the object recognition task. Importantly, by using (i) mice with a complete ablation of classical photoreceptors (*rd/rd cl*) [29,30] and (ii) *Opn4*^−/−^ mice with melanopsin deficiency but retaining rods/cones and pRGCs [16–20], we are able to examine which photoreceptors are required to modulate memory performance.

Here, we first examine if *rd/rd cl* and *Opn4*^−/−^ mice are capable of object discrimination. Based on previous findings [31–33], it is anticipated that these animals will not show any deficit in basic object discrimination, as this ability is not completely dependent on visual inputs. We then ask if recognition of the *visuospatial context* of an object (i.e. where an object is located) will require classical photoreceptors but not melanopsin, as rods and cones are primarily involved in IF vision. We then go on to examine how acute exposure to bright light will affect object recognition memory: would it facilitate or disrupt performance? In addition, will bright light exert differential effects when it is presented at the sample (i.e. encoding) versus test (i.e. retrieval) phase of the task (see [34])? Furthermore, is the effect of light on performance a consequence of elevated anxiety in response to bright light [12,21]? Crucially, will the modulatory effect of light on object recognition performance be attenuated in *rd/rd cl* or in *Opn4*^−/−^ mice? We hypothesize that any change in recognition memory performance in response to bright light will be dependent upon melanopsin [16–21], but not classical IF rods and cones.

## 2. Methods

### (a) Mice

All male mice were at least 12 weeks old at the time of behavioural testing. Animals were group housed with their littermates (two to five animals per cage), and were given *ad libitum* access to food and water. They were kept in a temperature-controlled colony with a 12-h:12-h light–dark cycle. Behavioural testing was always conducted during the light phase (3–6 h after light onset). Rodless–coneless (*rd/rd cl*) mice had a complete loss of rod and cone photoreceptors after 80 days of age [29,30]; these mice were maintained on a C3H background, and thus age-matched male C3H WT mice that did not carry the *rd* mutation were used as control subjects. Melanopsin-knockout mice were on a C57BL/6×129 background [20] and their *Opn4*^+/+^ WT littermates were used as control subjects; *Opn4*^−/−^ and *Opn4*^+/+^ mice were obtained from cross breeding heterozygous (*Opn4*^+/−^) mice. All behavioural procedures were performed in accordance with the United Kingdom Animals (Scientific Procedures) Act 1986 under Project Licence 30/2812 and Personal Licence I869292DB.

### (b) Spontaneous object recognition

**Figure 1.**
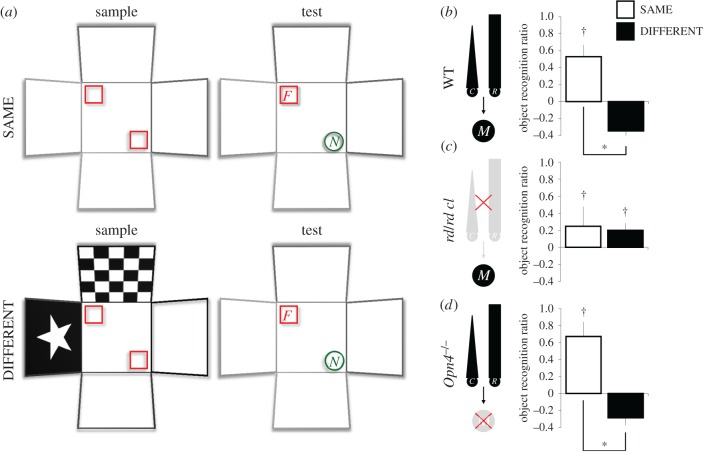
The effect of visual context change on object recognition performance depends on rods/cones but not melanopsin. The background visual context was manipulated (decorated or entirely white arena) but background irradiance was kept constant (100 lux). (*a*) Schematic shows the spontaneous object recognition task under the SAME (no context change) and DIFFERENT conditions (context change). In both conditions, a mouse was allowed to explore two identical replicates of an object (indicated by red squares) in the white arena for 10 min (the sample phase). After a 5 min delay, a novel object (indicated by green circles) was introduced and the animal was allowed to explore the familiar and novel objects for 2 min (the test phase). For animals in the SAME condition, both the sample and test phases were conducted in the white arena; but for animals in the DIFFERENT condition, the sample phase was conducted in a visually distinct arena. All other aspects of the task were identical under the two conditions. (*b–d*) Object recognition ratios in WT mice (both strains combined), *rd/rd cl* and *Opn4*^−/−^ mice, respectively. Performance in WT and *Opn4*^−/−^ mice was sensitive to a change in the background visual context. Recognition ratios were higher when the test was conducted in the same context than when it was conducted in a different context (SAME condition *n*s = 8 WT and 4 *Opn4*^−/−^ mice; DIFFERENT condition *n*s = 8 WT and 4 *Opn4*^−/−^ mice; (*b,d*)). No visual context effect was found in *rd/rd cl* mice (SAME condition *n* = 5; DIFFERENT condition *n* = 6; (*c*)); however, these mice could discriminate between novel and familiar objects (on the basis of non-visual features). In the diagrams in (*b–d*), R/C , rods/cones; M, melanopsin-expressing pRGCs; asterisk: significant effect of visual context (*p* < 0.005); dagger: significant object recognition performance (above zero; *p* < 0.05); error bars denote standard error of mean.

The standard object recognition task consisted of a sample phase, a delay period and a test phase [22–25]. On each trial, a mouse was allowed to explore (for 10 min) two identical replicates of an object (e.g. *F*_1_ and *F*_2_) in the sample phase; *F*_1_ and *F*_2_ were located at the top left and bottom right corners of the arena as shown in figure 1*a*. During the delay period, the animal was removed from the arena for 5 min, during which the walls and floor of the arena were cleaned with 50% ethanol. In the test phase, one replicate of *F* (e.g. *F*_2_) was replaced by a novel object (*N*), whereas replicate *F*_1_ was replaced by a third replicate of *F* (*F*_3_) that had never been explored by the animal. This eliminated the possibility that, during the test phase, the animal simply recognized and ignored its own odour traces left on the familiar object at pre-exposure. The animal was allowed to explore *F*_3_ and *N* for 2 min. Preference for the novel object (i.e. *N*) during this test phase provides evidence for memory of the familiar object (i.e. *F*). On each recognition trial, videos were recorded during the sample and test phases. Automated tracking was subsequently conducted with the ANY-maze software (version 4.5; Stoelting, Wood Dale, IL) as in previous studies [35–37].

### (c) The effect of visual context change on object recognition performance

To assess the sensitivity of object recognition performance to background visual context, half of the animals in each genotype were allocated to one of two conditions: SAME or DIFFERENT (figure 1*a*). In the SAME condition (four C3H WT, five *rd/rd cl*, four *Opn4*^+/+^ WT and four *Opn4*^−/−^), the white arena was used during both the sample and test phases (i.e. there was no change in the background context), and animals were given the standard object recognition task as described above (figure 1*a*, top panel). However, in the DIFFERENT condition (four C3H WT, six *rd/rd cl*, four *Opn4*^+/+^ WT and four *Opn4*^−/−^), there was a change in the background context between the sample and test phases (from the decorated arena to the white arena; figure 1*a*, bottom panel). Thus, the sample phases for the SAME and DIFFERENT conditions were carried out in different arenas, but the test phases for both conditions were carried out in the white arena. The light level was kept constant at 100 lux in the sample and test phases for both conditions. All other aspects of the task were identical between conditions. The types of object used and counterbalancing of object identities and locations are described in electronic supplementary material, figure S1 and electronic supplementary material, Methods.

We anticipated that mice with functional rods and cones (i.e. both strains of WT and *Opn4*^−/−^ mice) would encode and remember the background visual context in which an object was encountered, exhibiting differential levels of object novelty preference in the test phase of the SAME versus DIFFERENT conditions. More specifically, mice would show a stronger preference to explore the novel object in the SAME condition than in the DIFFERENT condition [38]. This context specificity of object novelty preference would reflect the formation and retrieval of a visual context–object association [39,40]. In *rd/rd cl* mice lacking classical photoreceptors, we anticipated that object novelty preference would be insensitive to a change in the background visual context, resulting in similar levels of performance in the SAME versus DIFFERENT conditions.

### (d) The modulatory effect of bright light on object recognition performance

**Figure 2.**
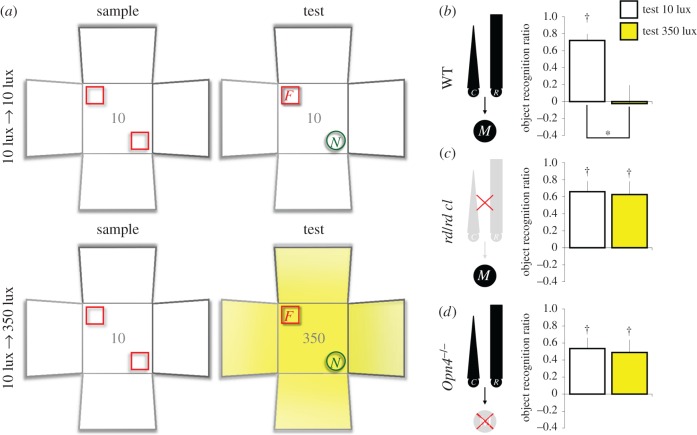
The modulatory effect of light on test performance depends on both rods/cones and melanopsin (sample phases at 10 lux). The background irradiance was manipulated at test (10 or 350 lux) but the visual context remained unchanged (white arena). (*a*) Schematic shows the spontaneous object recognition task under the 10 lux → 10 lux and 10 lux → 350 lux conditions. In both conditions, a mouse was allowed to explore two identical replicates of an object (indicated by red squares) in the white arena for 10 min in the sample phase. After a 5 min delay, a novel object (indicated by green circles) was introduced and the animal was allowed to explore the familiar and novel objects for 2 min in the test phase. For animals in the 10 lux → 10 lux condition, both the sample and test phases were performed under 10 lux, measured at the centre of the white arena. For animals in the 10 lux → 350 lux condition, the light level in the test phase was increased to 350 lux. All other aspects of the task were identical under the two conditions. (*b–d*) Object recognition ratios in WT mice, *rd/rd cl*, and *Opn4*^−/−^ mice, respectively. Performance in WT mice was sensitive to the background light level. Recognition ratios were higher when the test was given at 10 lux than when it was given at 350 lux (*n* = 22 in the 10-lux test; *n* = 12 in the 350-lux test; (*b*)). No effect of light on performance was found in *rd/rd cl* and *Opn4*^−/−^ mice (*n* = 8 per light condition in *rd/rd cl* mice; *n* = 6 per light condition in *Opn4*^−/−^ mice mice; (*c,d*)); however, both genotypes could discriminate between novel and familiar objects. In the diagrams in (*b–d*), R/C, rods/cones; M, melanopsin-expressing pRGCs; asterisk: significant effect of test irradiance (*p* < 0.005); dagger = significant object recognition performance (above zero; *p* < 0.05); error bars denote standard error of mean.

**Figure 3.**
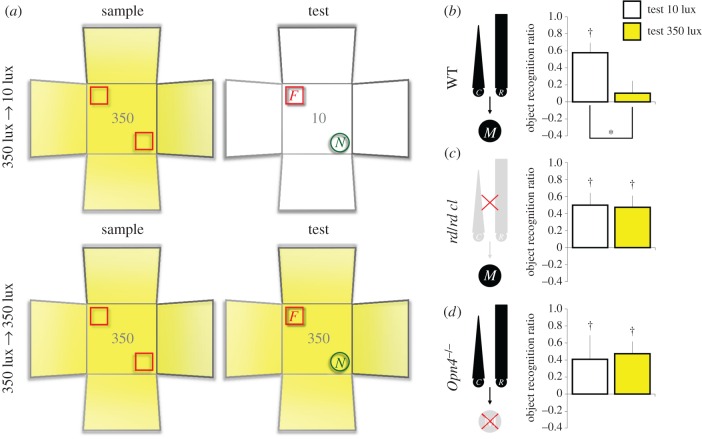
The modulatory effect of light on test performance depends on both rods/cones and melanopsin (sample phases at 350 lux). The background irradiance was manipulated at test (10 or 350 lux) but the visual context remained unchanged (white arena). (*a*) Schematic shows the spontaneous object recognition task under the 350 lux → 10 lux and 350 lux → 350 lux conditions. In both conditions, a mouse was allowed to explore two identical replicates of an object (indicated by red squares) in the white arena for 10 min in the sample phase. After a 5 min delay, a novel object (indicated by green circles) was introduced, and the animal was allowed to explore the familiar and novel objects for 2 min in the test phase. For animals in the 350 lux → 350 lux condition, both the sample and test phases were performed under 350 lux, measured at the centre of the white arena. For animals in the 350 lux → 10 lux condition, the light level in the test phase was reduced to 10 lux. All other aspects of the task were identical under the two conditions. (*b–d*) Object recognition ratios in WT mice, *rd/rd cl*, and *Opn4*^−/−^ mice, respectively. Performance in WT mice was sensitive to the background light level at test. Recognition ratios were higher when the test was given at 10 lux than when it was given at 350 lux (*n* = 10 in the 10-lux test; *n* = 19 in the 350-lux test; (*b*)). No effect of light on performance was found in *rd/rd cl* and *Opn4*^−/−^ mice (*n* = 5 per genotype in the 10 lux test; *n* = 10 per genotype in the 350 lux test; (*c,d*)); however, these mice could discriminate between novel and familiar objects. In the diagrams in (*b–d*), R/C, rods/cones; M, melanopsin-expressing pRGCs; asterisk: significant effect of test irradiance (*p* < 0.05); dagger: significant object recognition performance (above zero; *p* < 0.05); error bars denote standard error of mean.

To assess the sensitivity of object recognition performance to background irradiance during the sample and test phases, animals within each genotype were allocated to one of four (white LED) lighting conditions: (i) 10 lux → 10 lux (11 C3H WT, eight *rd/rd cl*, 11 *Opn4*^+/+^ WT and six *Opn4*^−/−^); (ii) 10 lux → 350 lux (six C3H WT, eight *rd/rd cl*, six *Opn4*^+/+^ WT and six *Opn4*^−/−^); (iii) 350 lux → 10 lux (five C3H WT, five *rd/rd cl*, five *Opn4*^+/+^ WT and five *Opn4*^−/−^) or (iv) 350 lux → 350 lux (10 C3H WT, 10 *rd/rd cl*, nine *Opn4*^+/+^ WT and 10 *Opn4*^−/−^). In all lighting conditions, animals were given the object recognition task in the white arena (figures 2 and 3). For some animals, the sample phases were conducted at 10 lux (10 lux → 10 lux and 10 lux → 350 lux conditions), whereas for the remaining animals, the sample phases were conducted at 350 lux (350 lux → 10 lux and 350 lux → 350 lux conditions). Before the test phase was conducted, the light level was increased to 350 lux for animals in the 10 lux → 350 lux condition (figure 2*a*, bottom panel), and it was decreased to 10 lux for animals 350 lux → 10 lux condition (figure 3*a*, top panel). For animals in the 10 lux → 10 lux and 350 lux → 350 lux conditions, the light intensity remained unchanged between the sample and test phases (figure 2a, top panel; figure 3*a*, bottom panel). All other aspects of the recognition task were identical among the four lighting conditions (see electronic supplementary material, figure S1 and electronic supplementary material, Methods for objects used and counterbalancing of object identities and locations).

By using a cross-over design, we could separate the modulatory effect of light at the time of encoding (which would be indicated by a main effect of sample irradiance) from the effect of light at the time of test (which would be indicated by a main effect of test irradiance). Alternatively, background irradiance might be used as a visual contextual cue to retrieve a memory of the previously encountered object (figure 1). If this were the case, WT mice would show better object recognition performance when the light levels in the sample and test phases were congruent than when they were incongruent, resulting in a sample irradiance × test irradiance interaction. We anticipated that any effect of light observed in WT mice would be attenuated in *Opn4*^−/−^ mice lacking melanopsin [16–21].

Our white LED light sources at 10 and 350 lux, measured at the centre of the floor of the arena, were equivalent to power values of 4 and 142 µW cm^−2^, respectively. Spectral power distributions of our white LED lights, which can be found in electronic supplementary material, S2, were measured with a radiometrically calibrated spectrophotometer (Ocean Optics, Oxford, UK) as in our previous studies [41]. The α-opic illuminance values for mouse ultraviolet-sensitive cone opsin (*λ*_max_ = 360 nm) [42], melanopsin (*λ*_max_ = 480 nm) [16,19], rhodopsin (*λ*_max_ = 498 nm) [43] and medium wavelength-sensitive cone opsin (*λ*_max_ = 508 nm) [44] were: 0.36, 11.60, 10.96 and 10.63, respectively, at 10 lux; 12.75, 405.88, 383.51 and 372.22, respectively, at 350 lux. These calculations were based on the rodent version of the irradiance toolbox [45], which used the photopigment complement of the mouse retina corrected for pre-receptoral spectral transmittance [46]. The Rodent Toolbox is freely available from www.ndcn.ox.ac.uk/team/stuart-peirson.

### (e) Other behavioural tasks: the optomotor response, elevated–plus maze and light–dark box tests

The optomotor response was assessed in the optokinetic drum [47–49] in order to examine visual acuity. The elevated–plus maze and light–dark box tests were used to assess the level of anxiety in each genotype. A detailed description of these behavioural tasks can be found in electronic supplementary material, Methods.

### (f) Data treatment and analyses

Recognition performance was expressed as a ratio, (*N* − *F*)/(*N* + *F*), where *N* and *F* represent total time spent in contact with novel versus familiar objects (or locations). Preference for novelty would give a ratio *greater than* zero, and preference for familiarity would give a ratio *less than* zero. A ratio equal to zero would suggest that an animal did not differentiate between novel and familiar objects (or spatial positions). Multiple analyses of variance (ANOVAs) with WT strain or genotype, and visual context or irradiance, as between-subjects factors were conducted on recognition ratios and total object exploration duration (which indicates overall exploratory activity). Multiple one-sample *t*-tests (two-tailed) were used to compare mean recognition ratios against the value of zero (i.e. no discrimination).

## 3. Results and discussion

### (a) Object recognition does not require visual input

We used novel and familiar objects that differed in multiple sensory modalities, allowing mice to solve the basic task in the absence of visual input. Consistent with this possibility, object recognition was significantly above zero in *rd/rd cl* mice (mean score ± standard error = 0.489 ± 0.119; *t*_7_ = 4.109, *p* < 0.01), and this was not significantly different from the level of performance observed in WT mice (mean score ± standard error = 0.482 ± 0.072; main effect of genotype: *F*_1,14_ = 0.003, *p* = 0.961). Similarly, melanopsin deficiency did not affect object recognition performance (WT versus *Opn4*^−/−^: 0.451 ± 0.093 versus 0.359 ± 0.117; *F*_1,14_ = 0.378, *p* = 0.549). The fact that object recognition is not affected by rod/cone loss or melanopsin deficiency is consistent with previous findings that WT animals are able to discriminate between different objects in the dark using non-visual cues [31,32].

### (b) Visuospatial recognition requires classical photoreceptors

By contrast, the object displacement task, which assesses animals' ability to discriminate between different spatial positions using different visual features on the walls of the arena (electronic supplementary material, figure S2*a*), was found to be dependent upon rods/cones but not melanopsin (main effect of genotype: *F*_2,37_ = 7.283, *p* < 0.005; *post hoc* least significant difference: *rd/rd cl* versus WT, *p* < 0.05; *rd/rd cl* versus *Opn4*^−/−^, *p* < 0.05; electronic supplementary material, figure S2*b*). The visual function and dysfunction of these mouse models were confirmed independently using the optokinetic drum, which assesses animals' ability to track rotating visual gratings of different spatial frequencies (electronic supplementary material, figure S2*c*). The loss of rods and cones, but not melanopsin, completely abolished the optomotor response (electronic supplementary material, figure S2*d*; see electronic supplementary material, Results and discussion).

### (c) A change in visual context impairs object recognition performance

Although object recognition does not require IF function, the ability to encode the background visual context in which objects are encountered is rod/cone-dependent. By using distinct visual contexts in the sample and test phases, we are able to determine animals' sensitivity to their visual environment (figure 1*a*). As anticipated, WT mice were sensitive to a change in the background context; this is indicated by a reduction in object recognition performance in animals that were tested in the different context compared with those that were tested in the same context (see also [38]). A two-way ANOVA conducted on the object recognition ratios for all WT mice, with strain (C3H versus *Opn4*^+/+^) and visual context (SAME versus DIFFERENT) as between-subjects factors, showed that mice that experienced a change in the visual context had lower recognition ratios than mice that did not experience such a change (main effect of visual context: *F*_1,12_ = 15.569, *p* < 0.005; figure 1*b*); however, there was no effect of context change on the level of object exploratory activity at test (*F*_1,12_ = 1.686, *p* = 0.218; electronic supplementary material, table S1). Notably, the visual response did not differ between the two strains of WT mice, as there was no strain × visual context interactive effect on recognition ratios (*F*_1,12_ = 0.503, *p* = 0.492). As there was no significant main effect of strain on test performance (*F*_1,12_ = 0.638, *p* = 0.440), the C3H and *Opn4*^+/+^ WT groups were combined, and one-sample *t*-tests showed that the mean recognition ratio in the SAME condition was significantly above zero (*t*_7_ = 3.826, *p* < 0.01), but the mean ratio in the DIFFERENT condition was not different from zero (*t*_7_ = −2.100, *p* = 0.074), further confirming that there was a reduction in object novelty preference after a change in the visual context.

#### (i) The visual context effect requires classical photoreceptors

As anticipated, in *rd/rd cl* mice object recognition performance was *insensitive* to the change in the background visual context (figure 1*c*). These mice displayed successful object recognition in both the SAME and DIFFERENT conditions. By contrast, mice lacking melanopsin showed differential levels of performance in the SAME versus DIFFERENT conditions (figure 1*d*), similar to that observed in WT mice.

More specifically, a between-subjects ANOVA conducted on the object recognition ratios, with genotype (WT combined, *rd/rd cl* and *Opn4*^−/−^) and visual context (SAME versus DIFFERENT) as factors, revealed a significant interaction (*F*_2,29_ = 4.648, *p* < 0.05), confirming that sensitivity to the change in the background context varied among genotypes. Like WT mice, *Opn4*^−/−^ mice that experienced a change in the visual context showed lower recognition ratios than mice that did not experience such a change (*F*_1,6_ = 25.477, *p* < 0.005; figure 1*d*). Furthermore, one sample *t*-tests showed that the mean recognition ratio in the SAME condition was significantly above zero (*t*_3_ = 3.965, *p* < 0.05), but the mean ratio in the DIFFERENT condition was not, comparable to the pattern observed in WT mice. By contrast, no visual context effect was found in *rd/rd cl* mice (*F*_1,9_ = 0.042, *p* = 0.842; figure 1*c*). Importantly, however, the mean recognition ratio (SAME and DIFFERENT groups combined) was significantly above zero (*t*_10_ = 2.039, *p* < 0.05), showing that these animals could still discriminate novel versus familiar objects on the basis of non-visual features. The different patterns of performance in WT and *Opn4*^−/−^ mice versus *rd/rd cl* mice were not owing to differential levels of exploratory activity, because there was no genotype difference in total time spent in object exploration during either the sample or test phase (electronic supplementary material, table S1).

### (d) Modulation of object recognition by light

Acute light exposure produces a dose-dependent elevation in different measures of arousal in mice, such as corticosterone [50], heart rate and locomotor activity [51], and also enhances conditioned fear responses [13]. However, we do not know how light would affect performance in the object recognition task: would it facilitate or disrupt performance? In addition, would light exert differential effects when it is presented at the sample versus test phase of the task?

We found that object recognition performance was disrupted when the test phase was conducted at 350 lux, *regardless* of the light level in the sample phase. A three-way ANOVA conducted on the object recognition ratios, with WT strain (C3H versus *Opn4*^+/+^), sample irradiance (10 versus 350 lux) and test irradiance (10 versus 350 lux) as between-subjects factors, showed that mice that experienced 350-lux tests had lower recognition ratios than mice that experienced 10-lux tests (main effect of test irradiance: *F*_1,55_ = 17.820, *p* < 0.005; electronic supplementary material, figure S3). However, there was no main effect of sample irradiance on performance (*F*_1,55_ = 0.003, *p* = 0.959; electronic supplementary material, figure S3), suggesting that exposure to a bright light at the time of test, rather than at the time of encoding, impaired performance. In addition, there was no sample irradiance × test irradiance interaction (*F*_1,55_ = 0.864, *p* = 0.357; electronic supplementary material, figure S3), implying that background irradiance *per se* is *not* a visual contextual cue that could aid the retrieval of object memory. Furthermore, the modulatory effect of light on object recognition did not differ between the two strains of WT mice, as there was no strain × test irradiance interaction (*F*_1,55_ = 0.848, *p* = 0.361), and there was no significant main effect of strain on performance (*F*_1,55_ = 2.607, *p* = 0.112). Consistent with the results from the ANOVA, one sample *t*-tests showed that mean recognition ratios in the 10-lux tests were significantly above zero (10 lux → 10 lux: *t*_21_ = 9.151, *p* < 0.0005; 350 lux → 10 lux: *t*_9_ = 4.978, *p* < 0.005; WT combined), but mean ratios in the 350-lux tests were not (10 lux → 350 lux: *t*_11_ = −0.110, *p* = 0.915; 350 lux → 350 lux: *t*_18_ = 0.719, *p* = 0.482; electronic supplementary material, figure S3).

A possible explanation for the absence of any object recognition performance at 350 lux is that the brighter light might have suppressed exploratory activity; similarly, it might have induced freezing or attempted escape responses that interfered with expression of object memory. However, the level of overall exploratory activity at test was not significantly different between 10 and 350 lux (electronic supplementary material, table S1).

#### (i) Modulation of object recognition by light is dependent on both melanopsin and classical photoreceptors

Which photoreceptors are required for the modulatory effect of light on object recognition performance? Strikingly, the effect of bright light was abolished in *rd/rd cl* as well as *Opn4*^−/−^ mice. A three-way ANOVA conducted on the object recognition ratios, with genotype (WT combined, *rd/rd cl* and *Opn4*^−/−^), sample irradiance (10 versus 350 lux) and test irradiance (10 versus 350 lux) as factors, revealed a main effect of test irradiance (*F*_1,109_ = 4.480, *p* < 0.05), as well as an interaction between genotype and test irradiance (*F*_2,109_ = 5.145, *p* < 0.01); no other effect was significant (all *p*s > 0.1). Unlike WT mice which showed differential levels of object recognition performance when tested at 10 versus 350 lux (figures 2*b* and 3*b*), there was no difference in recognition ratios at different light levels in *rd/rd cl* mice (simple main effect of test irradiance, figure 2*c*: *F*_1,14_ = 0.029, *p* = 0.867; figure 3*c*: *F*_1,13_ = 0.013, *p* = 0.912) or in *Opn4*^−/−^ mice (simple main effect of test irradiance, figure 2*d*: *F*_1,10_ = 0.053, *p* = 0.822; figure 3*d*: *F*_1,13_ = 0.052, *p* = 0.823). One-sample *t*-tests further confirmed that, in both *rd/rd cl* and *Opn4*^−/−^ mice mean recognition ratios in the 10- and 350-lux tests were all significantly above zero (figures 2 and 3). As before, there was no genotype difference in total time spent in object exploration during either the sample or test phase (electronic supplementary material, table S1).

#### (ii) Is the modulatory effect of light on performance related to anxiety?

A possible explanation for the modulatory effect of light on recognition performance in WT mice is that the brighter light might have induced a higher level of anxiety, which could lead to avoidance of novel objects (i.e. *neophobia*; [52]). Moreover, *rd/rd cl* and *Opn4*^−/−^ mice might have a *lower* level of anxiety than WT mice owing to attenuated (or altered) perception of light in the environment. This would then reduce or eliminate the neophobic response to novel objects observed under the brighter light. To test this hypothesis, we conducted two classic tests of anxiety―the elevated–plus maze and light–dark box tests―at 10 and 350 lux in the two strains of WT, *rd/rd cl* and *Opn4*^−/−^ mice. However, there was no difference in the level of open-arm avoidance in the elevated–plus maze or in light avoidance in the light–dark box between 10 and 350 lux in WT mice (electronic supplementary material, figures S4 and S5; see Results and Discussion in electronic supplementary material).

Interestingly, there were significant differences in performance among genotypes. In the elevated–plus maze *rd/rd cl* mice did not discriminate between the open versus closed arms, whereas WT and *Opn4*^−/−^ mice showed a significant preference for the closed arms (electronic supplementary material, figure S4*d*). Similarly, in the light–dark box, *rd/rd cl* mice showed a significant preference for the illuminated compartment of the apparatus, whereas WT and *Opn4*^−/−^ mice showed a significant preference for the dark compartment (electronic supplementary material, figure S5*e*). These data suggest that mice lacking classical photoreceptors had a *lower* level of state anxiety in these tasks than WT and *Opn4*^−/−^ mice (see also [53]). The fact that *rd/rd cl* and *Opn4*^−/−^ mice had significantly different levels of anxiety, and yet both were insensitive to the background light level during the test phase of the object recognition task, further suggests that the differential patterns of performance between WT versus *rd/rd cl* and *Opn4*^−/−^ mice were not directly related to anxiety.

## 4. General discussion

Acute exposure to bright light exerts various effects on physiology and behaviour in humans [1–4] as well as in nocturnal rodents [12,50,51]. Although the modulatory effects of light on brain network activity in humans are well demonstrated [5–8], the effects of light on cognitive performance are less clear, with varying results across different behavioural tasks [9–11,13–15,34]. Importantly, the retinal photoreceptors that mediate these responses have remained poorly defined. Evidence for the role of melanopsin in modulating human cognitive function is largely indirect [8], owing to the difficulty in specifically isolating pRGC contributions. Transgenic mouse models enable specific retinal pathways to be studied in isolation. However, to date, few studies have addressed the role of rods/cones and melanopsin in the regulation of memory performance in response to light. Here, we address this issue using the spontaneous object recognition task in mice lacking classical rod/cone photoreceptors (*rd/rd cl*) and mice lacking melanopsin-driven photoresponses (*Opn4*^−/−^).

### (a) Visuospatial recognition requires classical photoreceptors

Object recognition performance in WT mice was reduced by a change in the background visual context. It has been suggested that this effect of context shift on recognition performance is based upon an associative mechanism [39,40]. This visual associative process requires classical photoreceptors but not melanopsin. Without rods/cones, *rd/rd cl* mice were not able to encode the background visual context during the sample phase or detect any visual context shift at test, whereas classical photoreceptors alone in *Opn4*^−/−^ mice were sufficient for encoding an object's visuospatial context. These findings reflect the role of classical rod and cone photoreceptors in image formation.

### (b) Modulation of object recognition memory performance by light requires both melanopsin and classical photoreceptors

Significantly, we report for the first time that object recognition performance was abolished when the test phase was conducted under bright light, regardless of the light level in the sample phase. This demonstrates that exposure to a bright light at the time of test, rather than at the time of encoding, impaired performance. In particular, it should be emphasized that this was not an IF response reflecting a change in the background visual context (figure 1), because mice showed equivalent levels of object recognition performance when the light levels in the sample and test phases were congruent and when they were incongruent (figures 2 and 3). The modulatory effect of light on performance was completely abolished in mice lacking melanopsin, consistent with the role of pRGC photoresponses in irradiance detection [16–20]. More surprising is the finding that the effect of light on performance was also abolished in animals lacking classical photoreceptors, suggesting that this NIF response requires *both* melanopsin and classical photoreceptive systems. While other NIF responses, such as pupillary constriction, circadian entrainment and sleep induction, may be retained in *rd/rd cl* and *Opn4*^−/−^ mice [16–20,29,30], our data suggest that both melanopsin and rods/cones are required for normal pRGC function in order to mediate the effect of bright light on memory performance (see also [13], where light-induced enhancement in fear-conditioned-freezing performance requires both melanopsin and rods/cones).

Our findings also complement a growing body of evidence on how rod/cone and melanopsin systems interact in both IF and NIF responses to light. For example, it is known that rods and cones modulate certain NIF responses to light via inputs to OPN4-expressing pRGCs [54–57]. Furthermore, it has recently been reported that melanopsin may contribute to IF pathways [58–60]. Our findings suggest that the modulatory effect of light on behavioural performance depends upon integration of signals from melanopsin and classical photoreceptors. This integration may occur at the level of the retina, via interactions of rod/cone and melanopsin photoreceptors (electronic supplementary material, figure S6*a*), or in the brain, where information from IF and NIF pathways converges (electronic supplementary material, figure S6*b*). Evidence for retinal integration comes from a recent study showing that melanopsin is required for adaptation of the classical visual pathways [59]. Alternatively, the modulatory effect of light may involve downstream integration of signals from both IF and NIF pathways, which are known to project to different, but overlapping, brain targets. Evidence for this explanation comes from human imaging studies, which show that light results in activation of IF thalamic and NIF hypothalamic targets, which precede cortical activation [5–8]. However, owing to the effect of light on performance requiring both melanopsin and classical photoreceptors, differentiating between these two mechanisms is challenging, as any manipulation affecting IF or NIF pathways will abolish this NIF response.

### (c) Effects of bright light on anxiety

Very few studies have investigated the modulatory effect of light on performance in rodents except Warthen *et al.* [13], who demonstrated that acute exposure to light enhanced the conditioned-freezing response to a tone that had been paired with a mild electric shock, and that this effect of light was attenuated in *Pde6b^rd1/rd1^* mice lacking functional classical photoreceptors as well as in *Opn4*^−/−^ mice. Therefore, while acute light exposure enhanced performance in their fear conditioning task, it impaired performance in our non-aversive recognition task, suggesting that light may have different effects in non-aversive and aversive paradigms. It could be argued that the findings from Warthen *et al.* [13] were due to, at least in part, the effect of light on anxiety rather than on memory [12,21]. Notably, however, in our study we did not find any differential effect of 10-lux versus 350-lux light on anxiety in the elevated–plus maze or light–dark box test. Nevertheless, at present we cannot rule out the possibility that light levels higher than 350 lux might significantly elevate anxiety.

### (d) Effects of bright light on physiological arousal

Taken together with findings from previous studies employing different behavioural tasks [13–15], we suggest that the modulatory effect of light on memory performance in nocturnal rodents is *task-specific*: it facilitates fear conditioning performance [13,15], but disrupts object recognition performance as well as spatial navigation in the water maze task [14]. What is the possible explanation for these different modulatory effects of light on performance? Previous studies have shown that light levels can modulate the level of physiological and behavioural arousal [1–8,50,51]. Moreover, it has been established that the level of arousal determines performance, and that the exact form of the arousal–performance relationship varies (linear versus inverted-U), depending on the nature of the behavioural task [61,62]. This century-old principle, often known as the Yerkes–Dodson law [63], provides a potential framework for explaining the contrasting effects of light on performance in different behavioural tasks (although it should be emphasized that the Yerkes–Dodson law may not provide a complete explanation in certain situations, as demonstrated in a recent human study; see [11]).

Critically, it has been shown that high levels of arousal interfere with various forms of synaptic plasticity [64–67], and that they impair performance in tasks that rely on the hippocampal formation and interconnected cortical areas [62,64,68]. Furthermore, although arousal could affect both encoding of new information and subsequent memory retrieval, the magnitude of the effect at retrieval test is often greater than the effect during the acquisition phase [69]. This is entirely consistent with our observation that exposure to a bright light at the time of test, rather than at the time of encoding, impaired performance. By contrast, in behavioural tasks in which animals encode and retrieve a simple conditioned stimulus → unconditioned stimulus association, such as during eyeblink conditioning, high levels of arousal often enhance conditioned responses ([70–72]; but see [73]). This is consistent with the results from Warthen *et al.* [13] that conditioned emotional responses were enhanced by acute light exposure. Collectively, these differential effects of arousal on performance may reflect a shift in the control of behaviour to brain systems that mediate appropriate defensive responses in a given situation [74], given that high levels of arousal are often associated with increased danger.

## Supplementary Material

Electronic Supplementary Material 1: Figures S1–S6, Supplemental Methods, And Supplemental Results And Discussion

## Supplementary Material

Electronic Supplementary Material 2: Spectral Power Distributions Of White LEDs

## Supplementary Material

Electronic Supplementary Material 3: Table S1

## Supplementary Material

Electronic Supplementary Material 4: Data In Figures 1, 2, 3, S2, S3, S4, And S5

## Data accessibility

The data supporting this article can be found in electronic supplementary material, S4.
