# Supplementary material for: Modulation of recognition memory performance by light requires both melanopsin and classical photoreceptors
Source: Proc Biol Sci. 2016 Dec 28;283(1845):20162275. doi: 10.1098/rspb.2016.2275 (PMC5204172; doi:10.1098/rspb.2016.2275)
Supplement: Electronic Supplementary Material 1: Figures S1–S6, Supplemental Methods, And Supplemental Results And Discussion [file rspb20162275supp1.docx]

# Modulation of Recognition Memory Performance by Light Requires Both Melanopsin and Classical Photoreceptors

Shu K. E. Tam, Sibah Hasan, Steven Hughes, Mark W. Hankins, Russell G. Foster, David M. Bannerman and Stuart N. Peirson

# Electronic Supplementary Material 1: Figures *S1*–*S6*, Supplemental Methods, And Supplemental Results And Discussion


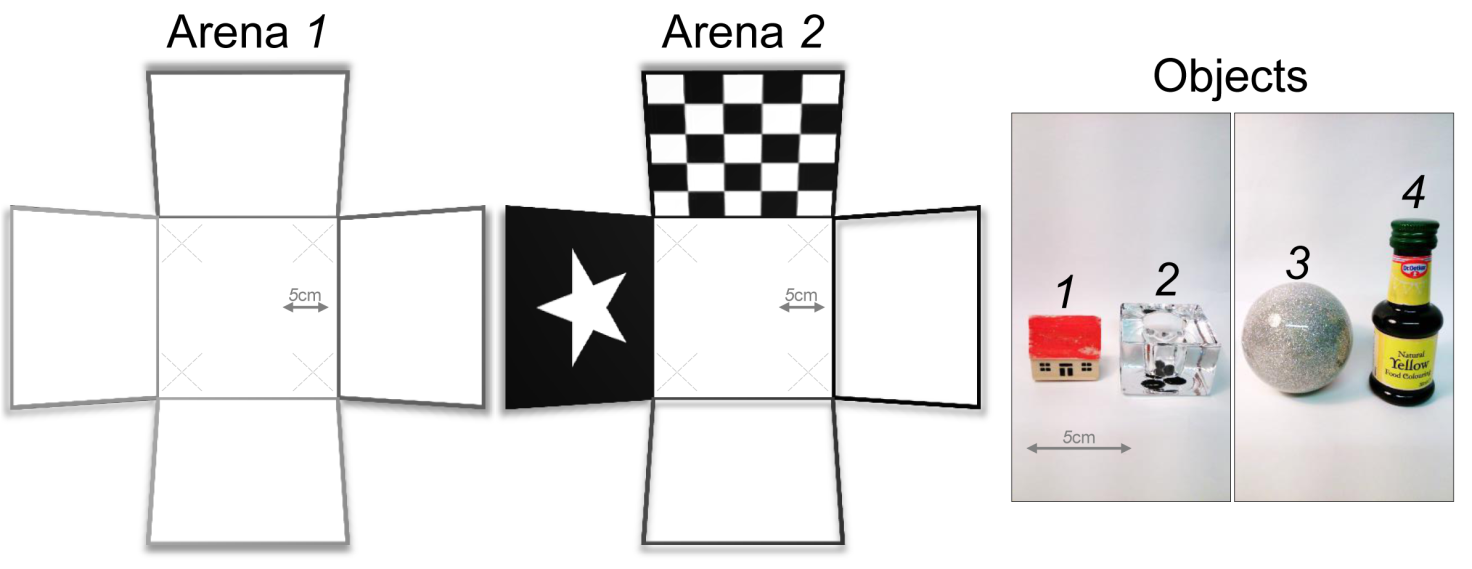


# Figure *S1*

## Arenas And Objects Used In The Recognition Experiments

**Arenas**: Both arenas were made of acrylic and had the same dimensions (20 cm × 20 cm × 20 cm). Arena *1* was white; arena *2* was transparent, but it was decorated with a checkerboard pattern and a white symmetrical five-point star (on a black background). **Objects**: Objects *1* and *2* were used in the visual context experiment; object *2* was used in the object displacement experiment; and objects *3* and *4* were used in the irradiance experiments. Object *1* was made of wood; object *2* was made of glass; objects *3* and *4* were made of plastic. There were four replicates of each object, so that different replicates of the same object could be presented in the sample and test phases. This eliminated the possibility that, during the test phase, the mouse simply recognised and ignored its own odour traces left on the familiar object at pre-exposure. The dimensions of these objects were―object *1*, length × width × height: 1.7 cm × 3.6 cm × 3.6 cm; object *2*, length × width × height: 4.4 cm × 4.5 cm × 3.0 cm; object *3*, diameter: 5.5 cm; and object *4*, base diameter × height: 3.3 cm × 8.8 cm. Assuming that the mouse’s eye is at the centre of the arena, each of these objects would subtend a visual angle of―object *1*, 14.5° (vertically) and 14.5° (horizontally); object *2*, 12.1° (vertically) and 18.0° (horizontally); object *3*, 22.0° (vertically) and 22.0° (horizontally); and object *4*, 34.6° (vertically) and 13.3° (horizontally). Visual angles in degrees are calculated from 2arctan[*k*/(2*d*)] × 180°/π, where *k* represents the height or width of an object and *d* the distance between the mouse’s eye and the object.


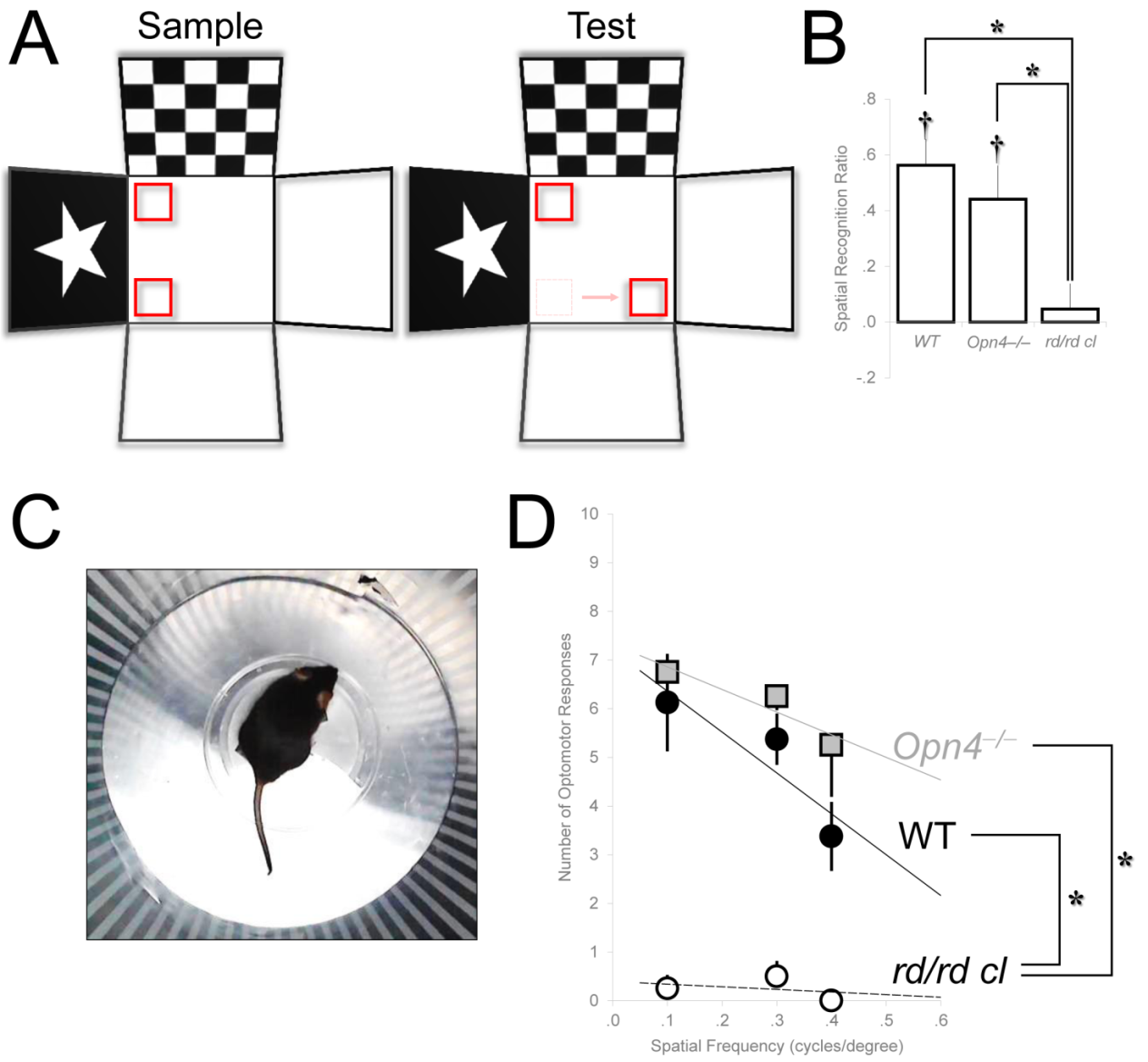


# Figure *S2*

## Recognition Performance In The Object Displacement Task And Optomotor Responses In The Optokinetic Drum

Panel **A**: Schematic showing the object displacement task. A mouse was allowed to explore two identical replicates of an object (indicated by red squares) in the decorated arena for 10 min (the *sample* phase). After a 5-min delay, one replicate was displaced to an adjacent corner and the animal was allowed to explore the two replicates for 2 min (the *test* phase). Panel **B**: Visuospatial recognition ratios in WT (12 C3H WT and 8 *Opn4*^+/+^ WT combined), *rd/rd cl* (*n*=12), and *Opn4*^−/−^ mice (*n*=8). Recognition performance in WT and *Opn4*^−/−^ mice was better than performance in *rd/rd cl* mice. Panels **C** and **D**: The number of optomotor responses per minute elicited by rotating visual gratings of different spatial frequencies (0.1, 0.3, and 0.4 cycles degree^−1^) was determined in the optokinetic drum (panel **C**). Optomotor responding in WT (4 C3H WT and 4 *Opn4*^+/+^ WT combined) and *Opn4*^−/−^ mice (*n*=4) was higher than that in *rd/rd cl* mice (*n*=4). In panels **B** and **D**, ***** = significant effect of Genotype (*p*<0.05); **†** = significant spatial novelty preference (above zero; *p*<0.01); error bars denote standard error of mean.

**
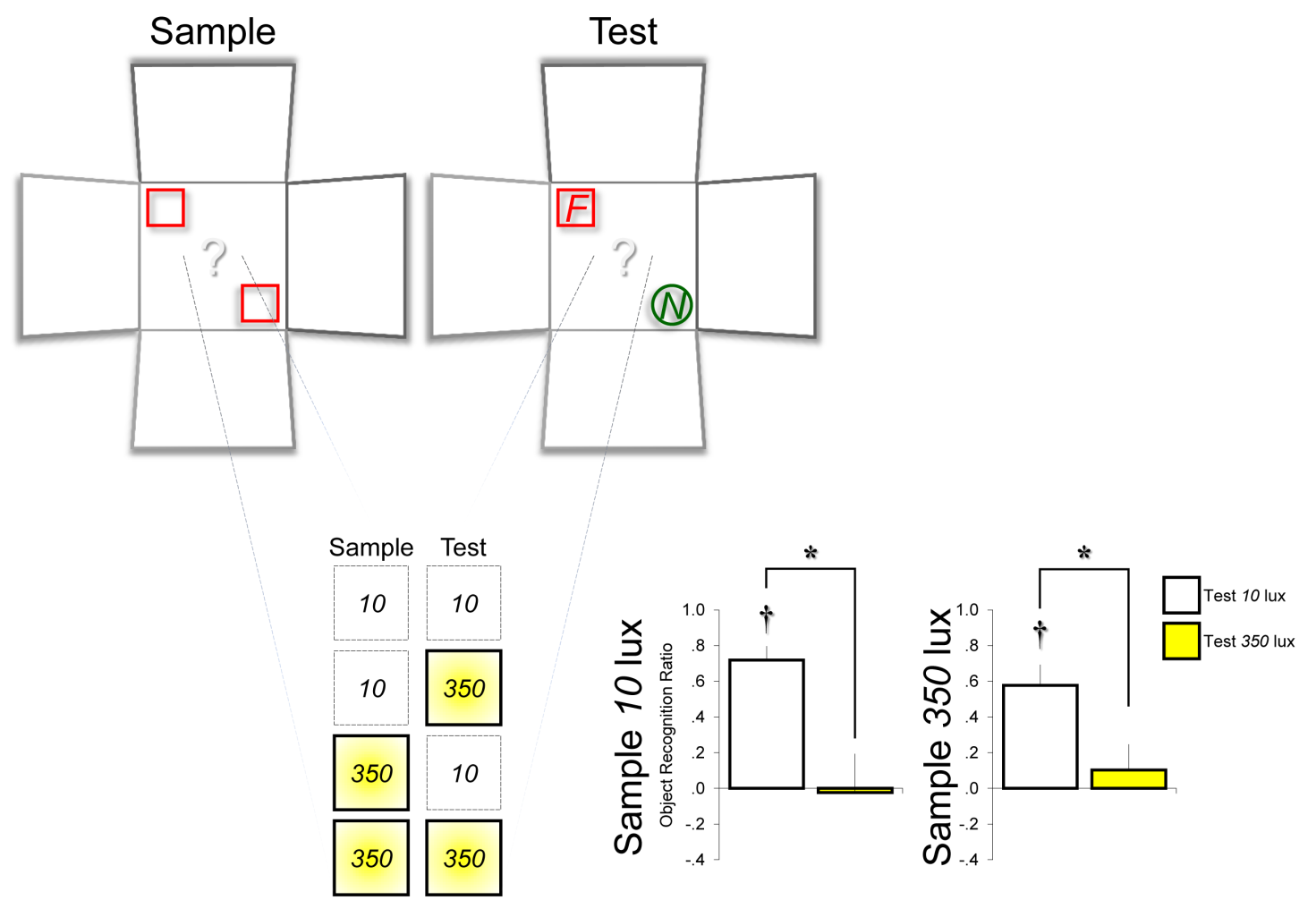
**

# Figure *S3*

## The Modulatory Effect Of Light On Object Recognition Performance In WT Mice

Bright light at test disrupted object recognition performance. The background light level in the sample and test phases was manipulated (10 or 350 lux) but the visual context remained unchanged (white arena). For some WT mice the sample phase was conducted at 10 lux, whereas for the remaining WT mice it was conducted at 350 lux. Within each of these two subgroups, some WT mice received the test under the same light level as in the sample phase (i.e. 10 lux → 10 lux, *n*=22; or 350 lux → 350 lux, *n*=19), whereas the remaining animals experienced either an increase or decrease in the background irradiance at test (i.e. 10 lux → 350 lux, *n*=12; or 350 lux → 10 lux, *n*=10); all other aspects of the task were identical under the different conditions. A mouse was allowed to explore two identical replicates of an object (indicated by red squares) in the white arena for 10 min in the sample phase. After a 5-min delay, a novel object (indicated by the green circle) was introduced and the animal was allowed to explore the familiar and novel objects for 2 min in the test phase. Performance in WT mice (C3H WT and *Opn4*^+/+^ WT combined) was sensitive to the light level at test. Recognition ratios were higher when the test was given at 10 lux than when it was given at 350 lux, *regardless* of the light level animals had experienced in the sample phase; ***** = significant effect of Test Irradiance (*p*<0.005); **†** = significant object recognition performance (above zero; *p*<0.0005); error bars denote standard error of mean.


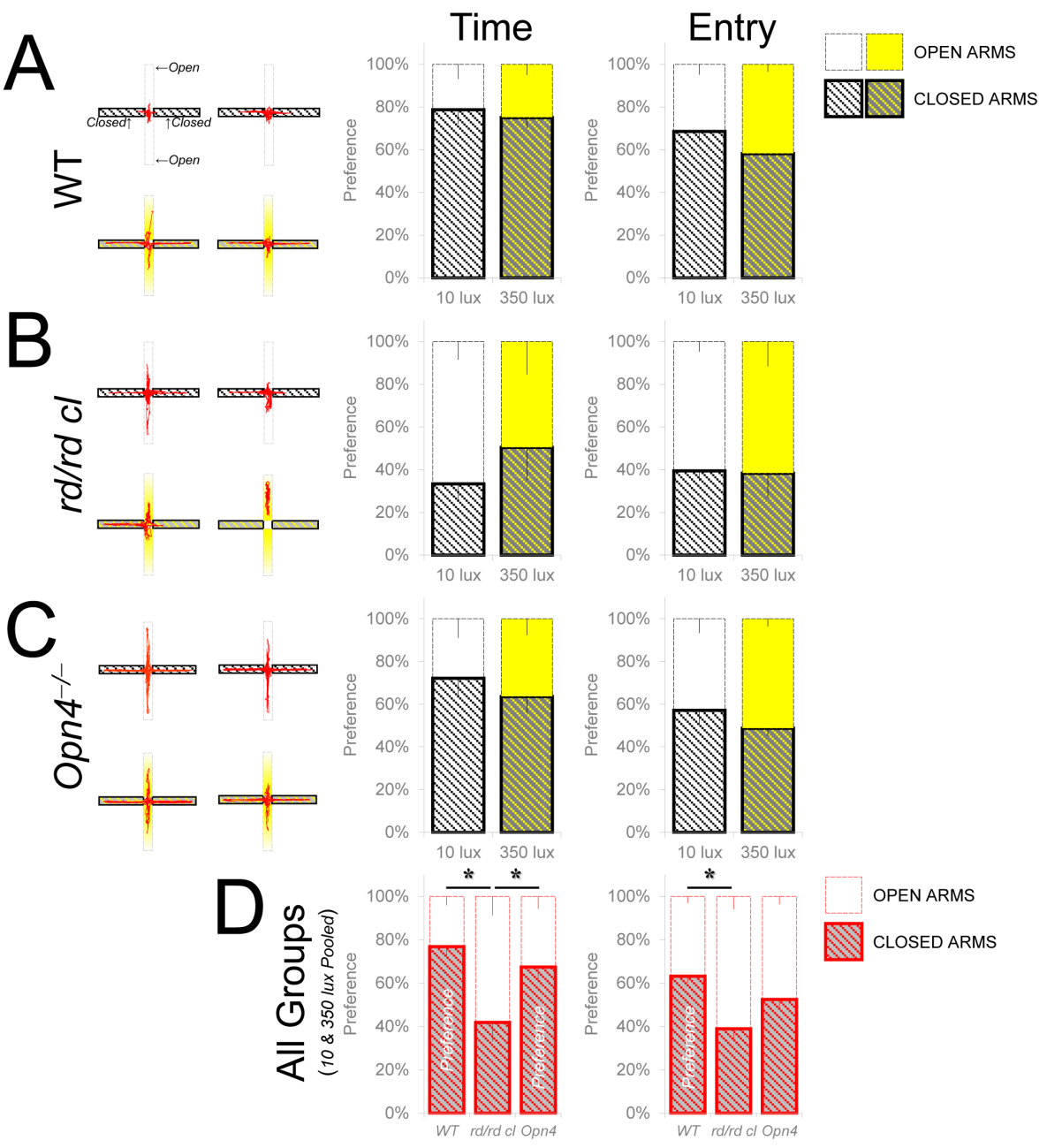


# Figure *S4*

## Anxiety Test I: The Elevated–plus Maze

Naïve mice (16 C3H WT, 10 *rd/rd cl*, 11 *Opn4*^+/+^ WT, and 9 *Opn4*^−/−^) were given an anxiety test at either 10 or 350 lux in the elevated–plus maze. The two arms at 12 and 6 o’clock positions were open and the two closed arms at 3 and 9 o’clock positions were surrounded by walls. The level of anxiety was determined from the percentage of time spent within, as well as entries into, the open and closed arms. Some examples of the track plots are shown on the *left* of panels **A**–**C**; the red traces indicate the path travelled under 10 lux (*white*) and 350 lux (*yellow*). There was no significant main effect of Irradiance (*p*s>0.20) or Irradiance × Genotype interaction (*p*s>0.35) on the two dependent measures. However, when data were pooled across light conditions (panel **D**), WT and *Opn4*^−/−^ mice (which preferred the *closed* arms) had higher levels of anxiety than *rd/rd cl* mice (which did not differentiate between open and closed arms). ***** = significant effect of Genotype (*p*<0.025); ***Preference*** = significant preference for the open or closed arms (*vs*. 50%; *p*<0.025); error bars denote standard error of mean.


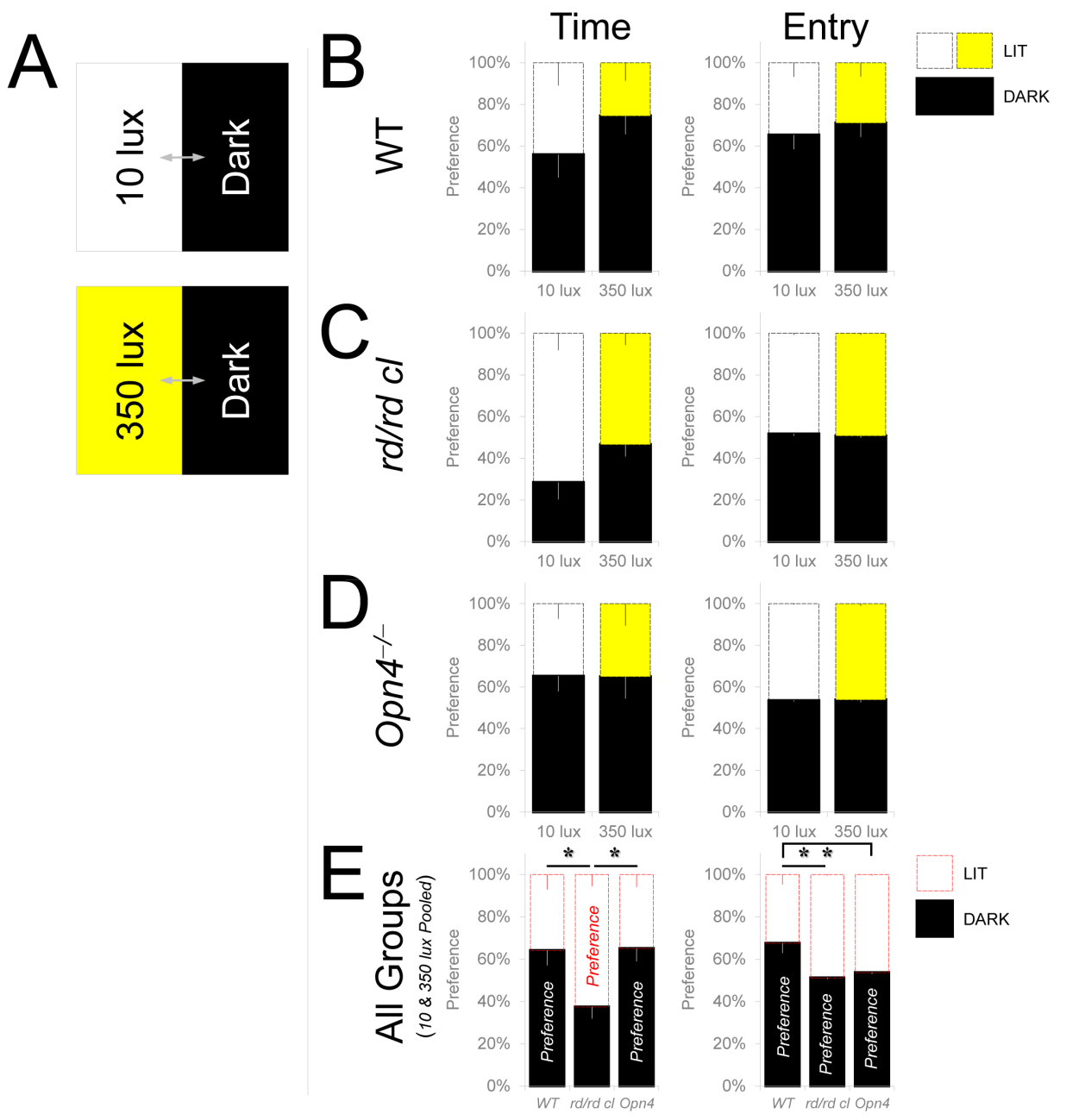


# Figure *S5*

## Anxiety Test II: The Light–dark Box

Naïve mice (10 C3H WT, 10 *rd/rd cl*, 9 *Opn4*^+/+^ WT, and 10 *Opn4*^−/−^) were given an anxiety test in the light–dark box, which comprised an illuminated compartment at either 10 or 350 lux and a dark compartment (panel **A**). Mice could move freely between compartments via a rectangular aperture. The level of anxiety was determined from the percentage of time spent within, as well as entries into, the illuminated and dark compartments (panels **B**–**E**). There was no significant main effect of Irradiance (*p*s>0.15) or Irradiance × Genotype interaction (*p*s>0.50) on the two dependent measures. However, when data were pooled across light conditions (panel **E**), WT and *Opn4*^−/−^ mice (which preferred the *dark* compartment) had higher levels of anxiety than *rd/rd cl* mice (which had a tendency to prefer the *illuminated* compartment). ***** = significant effect of Genotype (*p*<0.025); ***Preference*** = significant preference for the illuminated or dark compartment (*vs*. 50%; *p*<0.05); error bars denote standard error of mean.


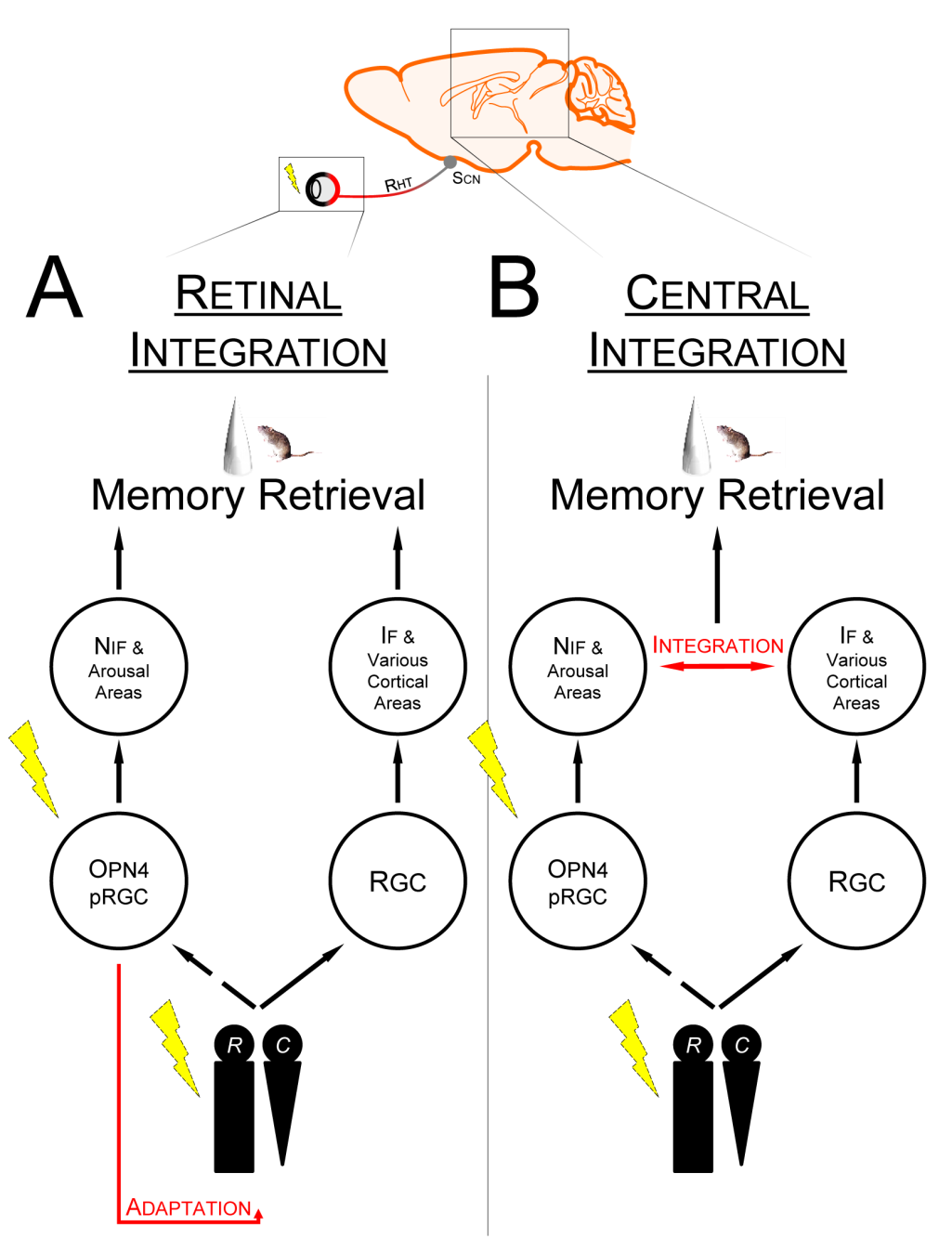


# Figure *S6*

## The Modulatory Effect Of Light On Recognition Performance Depends On Both Melanopsin And Classical Photoreceptors

The modulatory effect of light on object recognition performance depends upon integration of signals from melanopsin and classical photoreceptors. This integration may occur at the level of the retina via interactions of rod/cone and melanopsin photoreceptors, as shown in panel **A**, or in the brain where information from IF and NIF pathways converges, as shown in panel **B**. IF, image forming; NIF, non-image forming; OPN4 pRGC, melanopsin-expressing photosensitive retinal ganglion cells; *R*/*C*, rods/cones; RGC, reintal ganglion cells; RHT, retinohypothalamic tract; SCN, suprachiasmatic nucluei

# Supplemental Methods

## *(a) Object Recognition Arenas*

Two different open top arenas (20 cm × 20 cm × 20 cm) were used for recognition testing. One arena was made of transparent acrylic. To facilitate discrimination of the four corners, two distinct 20 cm × 20 cm wallpapers were attached to the outside of two walls (**Figure *S1***). One wallpaper had a checkerboard pattern with alternating 4 cm × 4 cm black and white squares; each square subtended a visual angle of 23° vertically and horizontally (assuming the mouse’s eye at the centre of the arena). The other wallpaper had a white symmetrical five-point star on a black background; the five-point star was drawn within a notational circle with a diameter of 14 cm, which subtended a visual angle of 70°. The other arena was made of white acrylic, and no wallpaper was attached to it (**Figure *S1***). Small pieces of hook-and-loop stickers (Rip ‘n’ Grip, Essex, UK) were adhered to the floor of the two arenas and to the bottom of all objects, so that objects could be affixed to the floor of the arena during recognition testing. A web camera for video recording was positioned 40 cm above the centre of the floor of the arena. White LED lights were positioned 40 cm above the floor of the arena, providing a light level of 100 lux in the visual context and visuospatial recognition experiments, and 10 or 350 lux in the irradiance experiment.

## *(b) Objects*

In a series of pilot studies with WT mice, we found that a variety of objects could be used as stimuli for spontaneous object recognition, providing that the size of the object was not too big for a mouse and that the objects to be discriminated differed in multiple sensory dimensions (e.g., colour, size, shape, and texture). Everyday objects such as bottles, light bulbs, and paperweights were among the variety of stimuli that were used in our laboratory for routine testing. For the current study, four different types of object were used: *1*) a glass candle holder [4.4 cm (length) × 4.5 cm (width) × 3.0 cm (height)]; *2*) a house-shaped wooden block with the top painted red [1.7 cm (length) × 3.6 cm (width) × 3.6 cm (height)]; *3*) a brown plastic bottle [3.3 cm (base diameter) × 8.8 cm (height)] with a green lid and two yellow labels; and *4*) a silver spherical ornament (5.5 cm in diameter) made of plastic. The dimensions of these four objects and the visual angles they subtended are summarised in the caption of **Figure *S1***. There were four replicates of each object, so that different replicates of the same object could be presented in the sample and test phases. This eliminated the possibility that, during the test phase, the subject simply recognised and ignored its own odour traces left on the familiar object at pre-exposure. Hereafter, the different replicates of an object (e.g., *F*) are indicated by *F*_1_, *F*_2_, *F*_3_, and *F*_4_.

## *(c) The Object Displacement Task*

The object displacement task [23] was similar to the standard object recognition task, except that the decorated arena was used for both sample and test phases, and that during the test phase animals were presented with two new replicates of the *familiar* object (*F*_3_ and *F* _4_). One replicate was displaced to an adjacent corner (e.g., from the bottom left corner to the bottom right corner), whereas the other replicate remained at the same location (e.g., top left; **Figure *S2A***). The glass candle holder (object *2* in **Figure *S1***) was used for all animals during the sample and test phases. The locations of the two replicates of the object during the sample phase, as well as the replicate that was selected for displacement at test, were counterbalanced within and between genotypes. More specifically for half of the animals within each genotype (6 C3H WT, 6 *rd/rd cl*, 4 *Opn4*^+/+^ WT, and 4 *Opn4*^−/−^), the replicates were located at the top left and bottom left corners during the sample phase; for the remaining half of the animals, the replicates were located at the top right and bottom right corners during the sample phase. In addition, for half of the animals within each of these counterbalanced subgroups (3 C3H WT, 3 *rd/rd cl*, 2 *Opn4*^+/+^ WT, and 2 *Opn4*^−/−^), the replicate located at the top was displaced at test (from top left to top right or *vice versa*), but the replicate at the bottom was not displaced. For the remaining animals, the replicate located at the bottom was displaced at test (from bottom left to bottom right or *vice versa*), but the replicate at the top was not displaced. We anticipated that mice with functional rods and cones (i.e. both strains of WT as well as *Opn4*^−/−^ mice) would encode the spatial positions of the sample objects with respect to the background visual stimuli, showing a preference to explore the object at the novel position at test; this is often taken as evidence for visuospatial recognition memory [23]. No spatial novelty preference would be expected in *rd/rd cl* mice lacking classical photoreceptors, because they would not be able to encode the background visual stimuli and hence the spatial positions of the objects relative to these cues.

## *(d) Counterbalancing Of Object Identities And Locations In The Visual Context And Irradiance Experiments*

In the visual context experiment (**Figure *1***), two types of object were used: the wooden block and glass candle holder (objects *1* and *2* in **Figure *S1***). They differed in multiple sensory dimensions and could be differentiated by non-visual as well as visual features. To take into account any potential bias towards a particular object or a certain part of the arena, the identities of familiar and novel objects, as well as their locations at test (top left *vs*. bottom right), were counterbalanced within and between genotypes and conditions. More specifically, for half of the animals in each condition (2 C3H WT, 2–3 *rd/rd cl*, 2 *Opn4*^+/+^ WT, and 2 *Opn4*^−/−^), the wooden block was used as the familiar object and the glass candle holder as the novel object at test; for the remaining animals, this arrangement was reversed. In addition, for half of the animals within each of these subgroups (1 C3H WT, 1–2 *rd/rd cl*, 1 *Opn4*^+/+^ WT, and 1 *Opn4*^−/−^), the novel object was located at the top left corner of the arena; for the remaining animals, it was located at the bottom right corner. In the irradiance experiments (**Figures *2*** and ***3***), the two types of object used―the bottle and spherical ornament (objects *3* and *4* in **Figure *S1***)―differed in multiple sensory dimensions as in the visual context experiment. The identities of familiar and novel objects and their locations at test were counterbalanced within and between genotypes and conditions, similar to that described above.

## *(e) The Optokinetic Drum*

The optomotor response test was conducted in the optokinetic drum [47–49], which was an apparatus with a cylindrical wall made of transparent acrylic. The cylindrical wall was 57 cm high with a diameter of 30 cm, and it was placed on a motorised plate that could be rotated in either a clockwise or an anticlockwise direction; the direction, speed, and duration of drum rotation were controlled by a computer. A circular platform with a diameter of 7 cm was located at the centre of the apparatus; it was raised 20 cm above the motorised plate. Attached to the circular platform was a Petri dish [8.8 cm (diameter) × 1.5 cm (height)], and a mouse was placed on the dish before the test. Wallpapers with alternating vertical black and white stripes (gratings) of different spatial frequencies (0.1, 0.3, and 0.4 cycles degree^−1^; assuming the mouse’s eye at the centre point of the cylindrical drum) were attached to the inner surface of the cylindrical wall. An overhead fluorescent lamp was positioned 80 cm above the motorised plate, providing a light level of about 100–150 lux on the circular platform; a web camera was attached to the lamp for video recording.

Four mice from each genotype were given three trials in the optokinetic drum, with an inter-trial interval of about 1 min. On each trial, a mouse was placed on the platform (**Figure *S2C***), and the drum was rotated in either a clockwise or an anticlockwise direction at a speed of 2 revolutions min^−1^ for a total duration of 112 s. Vertical gratings with different spatial frequencies, 0.1, 0.3, and 0.4 cycles degree^−1^, were used on each of the three trials. For half of the animals in each genotype (2 C3H WT, 2 *rd/rd cl*, 2 *Opn4*^+/+^ WT, and 2 *Opn4*^−/−^), the order of the grating stimuli presented was 0.4, 0.3, and 0.1 cycles degree^−1^ across the three trials. For the remaining half of the animals, the order of the grating stimuli presented was reversed (0.1, 0.3, and 0.4 cycles degree^−1^). In addition, within each of these subgroups, one animal per genotype had the drum rotated in a clockwise direction in all three trials; the remaining animal had the drum rotated in an anticlockwise direction. Videos were recorded during the three trials; from each video the experimenter counted the total number of optomotor responses, defined as a mouse’s head moving slowly in the same direction as the rotation of the drum while its body remained stationary [47,48].

## *(f) The Elevated*–*plus Maze*

The elevated–plus maze was a grey acrylic apparatus with a central platform area [5.0 cm × 5.0 cm] connected to four arms of identical dimensions [28.0 cm (length) × 5.0 cm (width)] that were perpendicular to each other. The two arms at 3 and 9 o’clock positions were open and the two arms at 6 and 12 o’clock positions were surrounded by grey acrylic walls that were 30 cm high; the walls were detachable from the arms. The surfaces of the central platform area and the arms were covered with white rubber linings. The whole apparatus was raised 49 cm above the floor by four aluminium legs attached to the base of the four arms. An overhead fluorescent lamp was positioned 65 cm above the central area, providing a light level of 330–350 lux on the central platform for some animals within each genotype (the 350 lux condition). For the remaining animals, several layers of neutral density (ND) filters were wrapped around the overhead lamp, so that the test was conducted at a lower light intensity of 8–10 lux (the 10 lux condition). A web camera was attached to the lamp for video recording.

Sixteen naïve C3H WT, 10 *rd/rd cl*, 11 *Opn4*^+/+^ WT, and 9 *Opn4*^−/−^ mice were tested in the elevated–plus maze. Half of the animals in each genotype (8 C3H WT, 5 *rd/rd cl*, 6 *Opn4*^+/+^ WT, and 5 *Opn4*^−/−^) received the test at 10 lux, whereas the remaining animals received the test at 350 lux. At the beginning of the trial, a mouse was placed in the central platform area, facing one of the open arms. The animal was allowed to explore the apparatus for a total duration of 5 min. Videos were recorded during the anxiety test, and automated tracking was subsequently conducted with the ANY-maze software, similar to that in the recognition experiments. The centre of the mouse was tracked in each video, and its position within the apparatus was determined on a second-by-second basis. The total number of entries into, and the amount of time spent within, the open and closed arms were recorded for every minute of the test.

## *(g) The Light*–*dark Box*

The light–dark box consisted of two detachable chambers of identical dimensions [26.0 cm (length) × 13.0 cm (width) × 30.0 cm (height)]. One chamber was made of transparent acrylic (the *illuminated* compartment), and the other was made of black acrylic (the *dark* compartment). The roofs of the transparent and black chambers were covered by transparent and black acrylic lids, respectively. The base of the transparent chamber was covered with white papers. The chambers were placed on a piece of transparent acrylic affixed with four anchors, which helped to align the two chambers and hold them in place. A mouse could move freely between the illuminated and dark compartments via a small aperture [4.7 cm (width) × 3.7 cm (height)]. For some animals within each genotype, an overhead fluorescent lamp was positioned 65 cm directly above the centre of the floor of the illuminated compartment, providing a light level of 330–350 lux in the 350 lux condition. For the remaining animals, several layers of ND filters were wrapped around the overhead lamp to provide a lower light intensity of 8–10 lux in the 10 lux condition. A web camera was attached to the lamp for video recording of a mouse’s behaviour in the illuminated compartment.

Ten naïve C3H WT, 10 *rd/rd cl*, 9 *Opn4*^+/+^ WT, and 10 *Opn4*^−/−^ mice were given an anxiety test in the light–dark box. For half of the animals in each genotype (5 C3H WT, 5 *rd/rd cl*, 4 *Opn4*^+/+^ WT, and 5 *Opn4*^−/−^), the light intensity in the illuminated compartment was 10 lux, whereas for the remaining animals the illuminated compartment was at 350 lux. At the beginning of the trial, a mouse was placed in the illuminated chamber, facing away from the rectangular aperture that connected the illuminated and dark compartments. The animal was allowed to explore the apparatus for a total duration of 5 min. The centre of the mouse was tracked in ANY-maze, and the total number of entries into, as well as the amount of time spent within, the illuminated chamber was recorded for every minute of the test.

# Supplemental Results And Discussion

## *(a) Visuospatial Recognition Requires Classical Photoreceptors*

In the *object displacement* task, the mouse explored two identical replicates of an object for 10 min in the sample phase within the decorated arena (which had a checkerboard pattern and a white star-shaped polygon on a black background on two walls; the remaining two walls were transparent; **Figure *S2A***). In the test phase, the animal was given two new replicates of the same object. One replicate of the object was now at a new position relative to the patterns on the walls, while the other replicate remained in the same position as in the sample phase (**Figure *S2A***); the animal was allowed to explore the two identical replicates for 2 min. All other aspects of the task were identical to the standard object recognition task. We anticipated that mice with functional rods and cones (both strains of WT and *Opn4*^−/−^ mice) would encode the spatial positions of the objects with respect to the background visual stimuli, showing a preference for the object at the novel position at test. This spatial novelty preference is often taken as evidence for visuospatial memory [23]. No spatial novelty preference would be expected in *rd/rd cl* mice, because they would not be able to encode the background visual stimuli (and hence the spatial positions of the objects relative to these cues).

Consistent with these predictions, visuospatial recognition was disrupted in *rd/rd cl* relative to WT and *Opn4*^−/−^ mice. There was a significant main effect of Genotype on spatial recognition ratios [*F*(2,37)=7.283,*p*<0.005]. *Post hoc* Least Significant Difference (LSD) tests found that recognition ratios in *rd/rd cl* mice were significantly lower than the recognition ratios in WT and *Opn4*^−/−^ mice (*p*s<0.05), but no difference was found between the latter two groups (*p*=0.441; **Figure *S2B***). In addition, recognition ratios in WT and *Opn4*^−/−^ mice were significantly above zero [both WT strains combined: *t*(19)=6.068,*p*<0.0005; *Opn4*^−/−^: *t*(7)=3.677,*p*<0.01], but recognition ratios in *rd/rd cl* mice were not [*t*(11)=0.512,*p*=0.619]. There was no genotype difference in total time spent in object exploration (**Table *S1*** in Electronic Supplementary Material 3).

## *(b) Optomotor Responding Requires Classical Photoreceptors*

To verify the image-forming deficit in *rd/rd cl* mice, we examined visual responses in an unrelated behavioural task―the optomotor response test―which assesses the mouse’s ability to track rotating visual gratings of different spatial frequencies (in an optokinetic drum; **Figure *S2C***). A split-plot ANOVA with Genotype (WT, *rd/rd cl*, and *Opn4*^−/−^) as a between-subjects factor and Spatial Frequency (0.1, 0.3, and 0.4 cycles degree^−1^) as a within-subjects factor found a significant main effect of Genotype on the number of optomotor responses [*F*(2,13)=25.392,*p*<0.0005]. *Post hoc* LSD tests found that *rd/rd cl* mice exhibited significantly fewer optomotor responses than WT and *Opn4*^−/−^ mice (*p*s<0.0005), but no difference was found between the latter two groups (*p*=0.169; **Figure *S2D***). This suggests that the loss of rods and cones, but not melanopsin, abolished the optomotor response―a finding consistent with previous studies [47–49]. Taken altogether, our data confirm that rods and cones, but not melanopsin, are required for stimulus-specific, image-forming visual responses.

## *(c) The Modulatory Effect Of Light On Recognition Performance Is Unrelated To Anxiety*

To test the hypothesis that the modulatory effect of light on object recognition performance is driven primarily by differential levels of anxiety under 10 and 350 lux, we conducted two classic tests of anxiety―the elevated–plus maze and light–dark box tests―at 10 and 350 lux in the two strains of WT, *rd/rd cl*, and *Opn4*^−/−^ mice. The level of anxiety was determined from the percentage of time spent within, as well as entries into, the open *vs*. closed arms in the elevated–plus maze, and the illuminated *vs*. dark compartments in the light–dark box.

Interestingly, in the elevated–plus maze test there was no main effect of Irradiance [TIME: *F*(1,40)=0.048,*p*=0.827; ENTRY: *F*(1,40)=1.685,*p*=0.202] or Irradiance × Genotype interaction [TIME: *F*(2,40)=0.995,*p*=0.379; ENTRY: *F*(2,40)=0.335,*p*=0.717]. However, there was a main effect of Genotype [TIME: *F*(2,40)=9.077,*p*<0.005; ENTRY: *F*(2,40)=9.500,*p*<0.0005]. This is due to that fact that WT and *Opn4*^−/−^ mice spent more time within the closed arms of the apparatus than *rd/rd cl* mice (*post hoc* LSD tests; *p*s<0.025). Multiple one-sample *t* tests (2-tailed) further confirmed that WT and *Opn4*^−/−^ mice showed a significant preference for the *closed* arms [both WT strains combined: TIME, *t*(27)=6.586,*p*<0.0005; ENTRY, *t*(27)=4.375,*p*<0.0005; *Opn4*^−/−^: TIME, *t*(9)=3.080,*p*<0.025; ENTRY, *t*(9)=0.683,*p*=0.514]. By contrast, *rd/rd cl* mice did not differentiate between the open or closed arms [TIME, *t*(9)=−0.937,*p*=0.373; ENTRY, *t*(9)=−1.919,*p*=0.087]. These findings are presented in **Figure *S4***.

Similar results were observed in the light–dark box test. There was no main effect of Irradiance [TIME: *F*(1,33)=2.153,*p*=0.152; ENTRY: *F*(1,33)=0.122,*p*=0.729] or Irradiance × Genotype interaction [TIME: *F*(2,33)=0.535,*p*=0.591; ENTRY: *F*(2,33)=0.215,*p*=0.808]. However, there was a main effect of Genotype [TIME: *F*(2,33)=4.660,*p*<0.025; ENTRY: *F*(2,33)=5.715,*p*<0.01]. This is due to that fact that WT and *Opn4*^−/−^ mice spent more time inside the dark compartment than *rd/rd cl* mice (*post hoc* LSD tests; *p*s<0.025). Multiple one-sample *t* tests (2-tailed) further confirmed that WT and *Opn4*^−/−^ mice showed a significant preference for the *dark* compartment [both WT strains combined: TIME, *t*(18)=2.231,*p*<0.05; ENTRY, *t*(18)=3.991,*p*<0.005; *Opn4*^−/−^: TIME, *t*(9)=2.517,*p*<0.05; ENTRY, *t*(9)=6.990,*p*<0.0005]. By contrast, *rd/rd cl* mice showed a significant preference for the *illuminated* compartment [TIME, *t*(9)=−2.296,*p*<0.05; although this was not the case for ENTRY, *t*(9)=2.429,*p*<0.05]. These findings are shown in **Figure *S5***.

Taken together, these data suggest that mice lacking classical photoreceptors had a *lower* level of state anxiety in these tasks than WT and *Opn4*^−/−^ mice. The fact that *rd/rd cl* and *Opn4*^−/−^ mice had significantly different levels of anxiety, and yet both were insensitive to the background light level during the test phase, implies that the different patterns of object recognition performance between WT *vs*. *rd/rd cl* and *Opn4*^−/−^ mice were not directly related to anxiety.
