# Supplementary material for: Modulation of recognition memory performance by light requires both melanopsin and classical photoreceptors
Source: Proc Biol Sci. 2016 Dec 28;283(1845):20162275. doi: 10.1098/rspb.2016.2275 (PMC5204172; doi:10.1098/rspb.2016.2275)
Supplement: Electronic Supplementary Material 2: Spectral Power Distributions Of White LEDs [file rspb20162275supp2.docx]

# Modulation of Recognition Memory Performance by Light Requires Both Melanopsin and Classical Photoreceptors

Shu K. E. Tam, Sibah Hasan, Steven Hughes, Mark W. Hankins, Russell G. Foster, David M. Bannerman and Stuart N. Peirson

# Electronic Supplementary Material 2: Spectral Power Distributions Of White LEDs

| nm | 350 lux | 10 lux |
| --- | --- | --- |
| **380** | 0.462 | 0.013 |
| **385** | 0.267 | 0.008 |
| **390** | 0.443 | 0.013 |
| **395** | 0.169 | 0.005 |
| **400** | 0.180 | 0.005 |
| **405** | 0.176 | 0.005 |
| **410** | 0.250 | 0.007 |
| **415** | 0.465 | 0.013 |
| **420** | 0.433 | 0.012 |
| **425** | 0.763 | 0.022 |
| **430** | 1.193 | 0.034 |
| **435** | 1.337 | 0.038 |
| **440** | 2.517 | 0.072 |
| **445** | 3.781 | 0.108 |
| **450** | 6.038 | 0.173 |
| **455** | 7.533 | 0.215 |
| **460** | 7.959 | 0.227 |
| **465** | 6.714 | 0.192 |
| **470** | 5.306 | 0.152 |
| **475** | 3.757 | 0.107 |
| **480** | 2.887 | 0.082 |
| **485** | 2.834 | 0.081 |
| **490** | 1.656 | 0.047 |
| **495** | 1.882 | 0.054 |
| **500** | 1.531 | 0.044 |
| **505** | 1.715 | 0.049 |
| **510** | 1.663 | 0.048 |
| **515** | 1.975 | 0.056 |
| **520** | 2.110 | 0.060 |
| **525** | 2.367 | 0.068 |
| **530** | 2.309 | 0.066 |
| **535** | 2.296 | 0.066 |
| **540** | 2.436 | 0.070 |
| **545** | 2.615 | 0.075 |
| **550** | 2.578 | 0.074 |
| **555** | 2.595 | 0.074 |
| **560** | 3.620 | 0.103 |
| **565** | 2.541 | 0.073 |
| **570** | 2.737 | 0.078 |
| **575** | 2.617 | 0.075 |
| **580** | 2.707 | 0.077 |
| **585** | 2.378 | 0.068 |
| **590** | 2.249 | 0.064 |
| **595** | 2.307 | 0.066 |
| **600** | 2.091 | 0.060 |
| **605** | 2.190 | 0.063 |
| **610** | 2.189 | 0.063 |
| **615** | 2.131 | 0.061 |
| **620** | 1.835 | 0.052 |
| **625** | 1.868 | 0.053 |
| **630** | 1.483 | 0.042 |
| **635** | 1.532 | 0.044 |
| **640** | 1.237 | 0.035 |
| **645** | 1.242 | 0.035 |
| **650** | 0.993 | 0.028 |
| **655** | 1.256 | 0.036 |
| **660** | 1.053 | 0.030 |
| **665** | 0.729 | 0.021 |
| **670** | 0.870 | 0.025 |
| **675** | 0.818 | 0.023 |
| **680** | 0.817 | 0.023 |
| **685** | 0.863 | 0.025 |
| **690** | 0.648 | 0.019 |
| **695** | 0.637 | 0.018 |
| **700** | 1.295 | 0.037 |
| **705** | 0.662 | 0.019 |
| **710** | 0.322 | 0.009 |
| **715** | 0.406 | 0.012 |
| **720** | 0.369 | 0.011 |
| **725** | 0.666 | 0.019 |
| **730** | 0.285 | 0.008 |
| **735** | 1.123 | 0.032 |
| **740** | 0.247 | 0.007 |
| **745** | 0.268 | 0.008 |
| **750** | 1.208 | 0.035 |
| **755** | 0.181 | 0.005 |
| **760** | 0.539 | 0.015 |
| **765** | 0.092 | 0.003 |
| **770** | 0.000 | 0.000 |
| **775** | 0.897 | 0.026 |
| **780** | 0.606 | 0.017 |
