# Supplementary material for: Modulation of recognition memory performance by light requires both melanopsin and classical photoreceptors
Source: Proc Biol Sci. 2016 Dec 28;283(1845):20162275. doi: 10.1098/rspb.2016.2275 (PMC5204172; doi:10.1098/rspb.2016.2275)
Supplement: Electronic Supplementary Material 3: Table S1 [file rspb20162275supp3.docx]

# Modulation of Recognition Memory Performance by Light Requires Both Melanopsin and Classical Photoreceptors

Shu K. E. Tam, Sibah Hasan, Steven Hughes, Mark W. Hankins, Russell G. Foster, David M. Bannerman and Stuart N. Peirson

# Electronic Supplementary Material 3: Table *S1*

# Table *S1*. Mean Rate Of Object Exploration (min^−1^) And Standard Error Of Mean In The Recognition Experiments

| Genotype | (*a*) Visual Context Experiment | | | | |  | (*b*) Object Displacement Experiment | | |
| --- | --- | --- | --- | --- | --- | --- | --- | --- | --- |
|  | *SAMPLE* | |  | *TEST* | |  | *SAMPLE* |  | *TEST* |
|  | SAME | DIFFERENT |  | SAME | DIFFERENT |  |  |  |  |
| C3H WT | 11.70 s (1.16 s) | 6.02 s (0.94 s) |  | 12.88 s (3.22 s) | 24.71 s (4.68 s) |  | 9.48 s (1.37 s) |  | 15.60 s (3.27 s) |
| *rd/rd cl* | 10.40 s (0.68 s) | 6.56 s (0.61 s) |  | 11.23 s (3.40 s) | 18.91 s (2.11 s) |  | 9.89 s (0.91 s) |  | 19.56 s (2.22 s) |
| *Opn4*^+/+^ WT | 11.48 s (3.09 s) | 5.62 s (1.65 s) |  | 16.51 s (5.54 s) | 16.50 s (4.47 s) |  | 6.57 s (0.69 s) |  | 7.37 s (2.72 s) |
| *Opn4*^−/−^ | 8.03 s (1.35 s) | 5.40 s (1.25 s) |  | 9.38 s (2.57 s) | 12.68 s (2.05 s) |  | 11.03 s (0.54 s) |  | 15.57 s (2.99 s) |
| Effect Of GENOTYPE^*^ | *F*(2,14)=1.459^†^ | *F*(2,15)=0.366^†^ |  | *F*(2,14)=0.720^†^ | *F*(2,15)=1.563^†^ |  | *F*(2,37)=2.009^†^ |  | *F*(2,37)=2.227^†^ |

NOTE: In the visual context experiment (panel *a*), there were 4 C3H WT, 5 *rd/rd cl*, 4 *Opn4*^+/+^ WT, and 4 *Opn4*^−/−^ mice in the SAME condition, and 4 C3H WT, 6 *rd/rd cl*, 4 *Opn4*^+/+^ WT, and 4 *Opn4*^−/−^ mice in the DIFFERENT condition. In the object displacement experiment (panel *b*), there were 12 C3H WT, 12 *rd/rd cl*, 8 *Opn4*^+/+^ WT, and 8 *Opn4*^−/−^ mice. The mean rate of object exploration in the *SAMPLE* phase was calculated by the total time spent in object exploration divided by the duration of the sample phase (i.e. 10 min). The mean rate of object exploration in the *TEST* phase was calculated by the sum of time spent in exploring novel and familiar objects (i.e. *N*+*F*) divided by the duration of the test phase (i.e. 2 min). These measures provide an indication of the level of exploratory activity. ^*^ Both strains of WT were combined in one-way between-subjects ANOVAs; ^†^ all *p*s>0.10; standard error of mean is shown in parentheses.

# Table *S1* (Continued)

| Genotype | (*c*) Irradiance Experiment (Sample Phase 10 lux) | | | | |  | (*d*) Irradiance Experiment (Sample Phase 350 lux) | | | | |
| --- | --- | --- | --- | --- | --- | --- | --- | --- | --- | --- | --- |
|  | *SAMPLE* | |  | *TEST* | |  | *SAMPLE* | |  | *TEST* | |
|  | 10 → 10 lux | 10 → 350 lux |  | 10 → 10 lux | 10 → 350 lux |  | 350 → 10 lux | 350 → 350 lux |  | 350 → 10 lux | 350 → 350 lux |
| C3H WT | 6.74 s (1.33 s) | 7.47 s (1.44 s) |  | 9.66 s (1.80 s) | 18.22 s (7.68 s) |  | 18.78 s (1.72 s) | 13.02 s (1.99 s) |  | 18.32 s (3.63 s) | 10.24 s (3.99 s) |
| *rd/rd cl* | 7.60 s (0.71 s) | 7.07 s (1.26 s) |  | 11.80 s (2.88 s) | 9.37 s (2.20 s) |  | 16.21 s (1.71 s) | 15.81 s (2.27 s) |  | 17.25 s (0.96 s) | 13.56 s (1.06 s) |
| *Opn4*^+/+^ WT | 7.79 s (1.70 s) | 7.36 s (1.38 s) |  | 12.45 s (5.11 s) | 12.70 s (3.23 s) |  | 8.58 s (2.53 s) | 8.41 s (2.02 s) |  | 9.30 s (2.52 s) | 6.36 s (1.98 s) |
| *Opn4*^−/−^ | 5.18 s (1.11 s) | 5.94 s (1.05 s) |  | 6.90 s (1.84 s) | 8.14 s (1.38 s) |  | 10.56 s (1.51 s) | 11.24 s (1.08 s) |  | 9.84 s (2.66 s) | 12.38 s (2.08 s) |
| Effect Of GENOTYPE^*^ | *F*(2,23)=1.278^†^ | *F*(2,23)=0.419^†^ |  | *F*(2,23)=0.737^†^ | *F*(2,23)=1.324^†^ |  | *F*(2,17)=1.237^†^ | *F*(2,36)=2.385^†^ |  | *F*(2,17)=1.552^†^ | *F*(2,36)=1.665^†^ |
| Effect Of TEST IRRADIANCE^**^ | WT:*F*(1,22)=0.744^†^; *rd/rd cl*:*F*(1,14)=0.666^†^; *Opn4*^−/−^:*F*(1,10)=0.665^†^ | | | | |  | WT:*F*(1,27)=2.163^†^; *rd/rd cl*:*F*(1,13)=1.227^†^; *Opn4*^−/−^:*F*(1,13)=0.561^†^ | | | | |

NOTE: In the irradiance experiment (sample phase 10 lux; panel *c*), there were 11 C3H WT, 8 *rd/rd cl*, 11 *Opn4*^+/+^ WT, and 6 *Opn4*^−/−^ mice in the 10 → 10 lux condition, and 6 C3H WT, 8 *rd/rd cl*, 6 *Opn4*^+/+^ WT, and 6 *Opn4*^−/−^ mice in the 10 → 350 lux condition. In the irradiance experiment (sample phase 350 lux; panel *d*), there were 5 C3H WT, 5 *rd/rd cl*, 5 *Opn4*^+/+^ WT, and 5 *Opn4*^−/−^ mice in the 350 → 10 lux condition, and 10 C3H WT, 10 *rd/rd cl*, 9 *Opn4*^+/+^ WT, and 10 *Opn4*^−/−^ mice in the 350 → 350 lux condition. The mean rate of object exploration in the *SAMPLE* phase was calculated by the total time spent in object exploration divided by the duration of the sample phase (i.e. 10 min). The mean rate of object exploration in the *TEST* phase was calculated by the sum of time spent in exploring novel and familiar objects (i.e. *N*+*F*) divided by the duration of the test phase (i.e. 2 min). These measures provide an indication of the level of exploratory activity. ^*^ Both strains of WT were combined in one-way between-subjects ANOVAs; ^**^ Two-way split-plot ANOVAs (Irradiance Condition × Phase) were conducted for each group; ^†^ all *p*s>0.10; standard error of mean is shown in parentheses.
