# Supplementary material for: Modulation of recognition memory performance by light requires both melanopsin and classical photoreceptors
Source: Proc Biol Sci. 2016 Dec 28;283(1845):20162275. doi: 10.1098/rspb.2016.2275 (PMC5204172; doi:10.1098/rspb.2016.2275)
Supplement: Electronic Supplementary Material 4: Data In Figures 1, 2, 3, S2, S3, S4, And S5 [file rspb20162275supp4.docx]

# Modulation of Recognition Memory Performance by Light Requires Both Melanopsin and Classical Photoreceptors

Shu K. E. Tam, Sibah Hasan, Steven Hughes, Mark W. Hankins, Russell G. Foster, David M. Bannerman and Stuart N. Peirson

# Electronic Supplementary Material 4: Data In Figures *1*, *2*, *3*, *S2*, *S3*, *S4*, And *S5*

| Figure 1 | |  |  |  |  |
| --- | --- | --- | --- | --- | --- |
| Genotype | Context | Sample | Test (New) | Test (Old) | Ratio |
| C3H WT | Same | 13.000 | 6.600 | 3.733 | 0.277 |
| C3H WT | Same | 14.305 | 17.133 | 5.233 | 0.532 |
| C3H WT | Same | 9.525 | 8.000 | 0.000 | 1.000 |
| C3H WT | Same | 9.960 | 10.667 | 0.133 | 0.975 |
| C3H WT | Different | 3.930 | 5.067 | 10.633 | -0.355 |
| C3H WT | Different | 6.957 | 7.000 | 20.567 | -0.492 |
| C3H WT | Different | 8.120 | 14.133 | 4.867 | 0.488 |
| C3H WT | Different | 5.070 | 0.400 | 36.167 | -0.978 |
| rd/rd cl | Same | 12.535 | 3.067 | 7.600 | -0.425 |
| rd/rd cl | Same | 11.485 | 15.533 | 0.100 | 0.987 |
| rd/rd cl | Same | 9.415 | 6.267 | 3.267 | 0.315 |
| rd/rd cl | Same | 9.605 | 0.000 | 0.000 | 0.000 |
| rd/rd cl | Same | 8.975 | 13.800 | 6.533 | 0.357 |
| rd/rd cl | Different | 4.465 | 13.367 | 11.300 | 0.084 |
| rd/rd cl | Different | 5.095 | 16.333 | 7.600 | 0.365 |
| rd/rd cl | Different | 6.490 | 5.600 | 7.067 | -0.116 |
| rd/rd cl | Different | 7.655 | 12.633 | 6.633 | 0.311 |
| rd/rd cl | Different | 8.065 | 7.300 | 5.667 | 0.126 |
| rd/rd cl | Different | 7.595 | 14.233 | 5.700 | 0.428 |
| Opn4 WT | Same | 5.881 | 4.267 | 4.300 | -0.004 |
| Opn4 WT | Same | 6.494 | 4.533 | 1.233 | 0.572 |
| Opn4 WT | Same | 15.810 | 15.200 | 13.400 | 0.063 |
| Opn4 WT | Same | 17.750 | 21.000 | 2.100 | 0.818 |
| Opn4 WT | Different | 3.285 | 3.567 | 6.033 | -0.257 |
| Opn4 WT | Different | 2.530 | 8.500 | 6.967 | 0.099 |
| Opn4 WT | Different | 9.580 | 3.933 | 25.467 | -0.732 |
| Opn4 WT | Different | 7.070 | 2.733 | 8.800 | -0.526 |
| Opn4 KO | Same | 8.878 | 7.767 | 0.800 | 0.813 |
| Opn4 KO | Same | 4.020 | 3.700 | 2.433 | 0.207 |
| Opn4 KO | Same | 9.775 | 4.950 | 1.000 | 0.664 |
| Opn4 KO | Same | 9.461 | 16.867 | 0.000 | 1.000 |
| Opn4 KO | Different | 3.095 | 3.300 | 3.967 | -0.092 |
| Opn4 KO | Different | 3.875 | 4.933 | 7.300 | -0.193 |
| Opn4 KO | Different | 8.675 | 4.150 | 10.050 | -0.415 |
| Opn4 KO | Different | 5.955 | 4.750 | 12.250 | -0.441 |

| Figure 2 | |  |  |  |  |  |
| --- | --- | --- | --- | --- | --- | --- |
| Genotype | Sample Lux | Test Lux | Sample | Test (New) | Test (Old) | Ratio |
| C3H WT | 10.000 | 10.000 | 10.920 | 11.100 | 0.050 | 0.991 |
| C3H WT | 10.000 | 10.000 | 10.825 | 7.067 | 2.367 | 0.498 |
| C3H WT | 10.000 | 10.000 | 5.550 | 8.333 | 0.000 | 1.000 |
| C3H WT | 10.000 | 10.000 | 5.195 | 15.200 | 0.000 | 1.000 |
| C3H WT | 10.000 | 10.000 | 4.345 | 2.000 | 0.033 | 0.967 |
| C3H WT | 10.000 | 10.000 | 3.630 | 11.800 | 0.000 | 1.000 |
| Opn4 WT | 10.000 | 10.000 | 6.135 | 5.900 | 1.100 | 0.686 |
| Opn4 WT | 10.000 | 10.000 | 14.735 | 8.400 | 15.500 | -0.297 |
| Opn4 WT | 10.000 | 10.000 | 8.610 | 23.500 | 8.700 | 0.460 |
| Opn4 WT | 10.000 | 10.000 | 9.605 | 5.800 | 0.300 | 0.902 |
| Opn4 WT | 10.000 | 10.000 | 3.255 | 1.333 | 0.167 | 0.778 |
| Opn4 WT | 10.000 | 10.000 | 4.405 | 3.967 | 0.033 | 0.983 |
| C3H WT | 10.000 | 10.000 | 12.890 | 9.250 | 0.000 | 1.000 |
| C3H WT | 10.000 | 10.000 | 9.850 | 6.450 | 4.800 | 0.147 |
| C3H WT | 10.000 | 10.000 | 7.320 | 6.150 | 0.100 | 0.968 |
| C3H WT | 10.000 | 10.000 | 12.860 | 47.450 | 0.000 | 1.000 |
| C3H WT | 10.000 | 10.000 | 17.290 | 26.350 | 0.000 | 1.000 |
| Opn4 WT | 10.000 | 10.000 | 3.940 | 4.950 | 0.150 | 0.941 |
| Opn4 WT | 10.000 | 10.000 | 13.050 | 14.750 | 6.400 | 0.395 |
| Opn4 WT | 10.000 | 10.000 | 7.280 | 3.900 | 0.950 | 0.608 |
| Opn4 WT | 10.000 | 10.000 | 5.510 | 12.550 | 2.150 | 0.707 |
| Opn4 WT | 10.000 | 10.000 | 10.780 | 7.600 | 6.650 | 0.067 |
| C3H WT | 10.000 | 350.000 | 11.170 | 7.667 | 2.733 | 0.474 |
| C3H WT | 10.000 | 350.000 | 12.280 | 2.400 | 2.667 | -0.053 |
| C3H WT | 10.000 | 350.000 | 3.570 | 4.600 | 0.000 | 1.000 |
| C3H WT | 10.000 | 350.000 | 6.405 | 14.633 | 0.000 | 1.000 |
| C3H WT | 10.000 | 350.000 | 6.910 | 0.000 | 54.733 | -1.000 |
| C3H WT | 10.000 | 350.000 | 4.465 | 0.000 | 19.867 | -1.000 |
| Opn4 WT | 10.000 | 350.000 | 10.530 | 0.600 | 4.100 | -0.745 |
| Opn4 WT | 10.000 | 350.000 | 11.990 | 14.900 | 5.500 | 0.461 |
| Opn4 WT | 10.000 | 350.000 | 6.400 | 1.600 | 10.100 | -0.726 |
| Opn4 WT | 10.000 | 350.000 | 5.550 | 17.600 | 4.900 | 0.564 |
| Opn4 WT | 10.000 | 350.000 | 6.915 | 3.100 | 10.700 | -0.551 |
| Opn4 WT | 10.000 | 350.000 | 2.790 | 2.000 | 1.100 | 0.290 |
| rd/rd cl | 10.000 | 10.000 | 7.950 | 8.267 | 1.133 | 0.759 |
| rd/rd cl | 10.000 | 10.000 | 10.733 | 8.500 | 2.000 | 0.619 |
| rd/rd cl | 10.000 | 10.000 | 4.660 | 26.467 | 1.167 | 0.916 |
| rd/rd cl | 10.000 | 10.000 | 9.583 | 19.150 | 1.800 | 0.828 |
| rd/rd cl | 10.000 | 10.000 | 6.250 | 7.367 | 0.000 | 1.000 |
| rd/rd cl | 10.000 | 10.000 | 8.345 | 2.200 | 2.967 | -0.148 |
| rd/rd cl | 10.000 | 10.000 | 7.580 | 3.900 | 0.950 | 0.608 |
| rd/rd cl | 10.000 | 10.000 | 5.685 | 7.200 | 1.333 | 0.688 |
| rd/rd cl | 10.000 | 350.000 | 11.150 | 2.700 | 0.000 | 1.000 |
| rd/rd cl | 10.000 | 350.000 | 13.075 | 9.650 | 6.750 | 0.177 |
| rd/rd cl | 10.000 | 350.000 | 2.560 | 18.000 | 0.000 | 1.000 |
| rd/rd cl | 10.000 | 350.000 | 3.370 | 14.833 | 0.000 | 1.000 |
| rd/rd cl | 10.000 | 350.000 | 5.740 | 1.167 | 1.133 | 0.014 |
| rd/rd cl | 10.000 | 350.000 | 6.325 | 5.333 | 0.867 | 0.720 |
| rd/rd cl | 10.000 | 350.000 | 6.840 | 3.267 | 2.633 | 0.107 |
| rd/rd cl | 10.000 | 350.000 | 7.500 | 8.500 | 0.100 | 0.977 |
| Opn4 KO | 10.000 | 10.000 | 5.060 | 9.700 | 2.800 | 0.552 |
| Opn4 KO | 10.000 | 10.000 | 1.550 | 6.900 | 0.400 | 0.890 |
| Opn4 KO | 10.000 | 10.000 | 4.690 | 8.300 | 2.300 | 0.566 |
| Opn4 KO | 10.000 | 10.000 | 9.855 | 0.000 | 0.000 | 0.000 |
| Opn4 KO | 10.000 | 10.000 | 4.125 | 2.700 | 1.167 | 0.397 |
| Opn4 KO | 10.000 | 10.000 | 5.815 | 6.433 | 0.700 | 0.804 |
| Opn4 KO | 10.000 | 350.000 | 6.480 | 10.000 | 4.400 | 0.389 |
| Opn4 KO | 10.000 | 350.000 | 5.575 | 5.600 | 0.000 | 1.000 |
| Opn4 KO | 10.000 | 350.000 | 2.885 | 6.300 | 1.600 | 0.595 |
| Opn4 KO | 10.000 | 350.000 | 3.195 | 4.100 | 1.600 | 0.439 |
| Opn4 KO | 10.000 | 350.000 | 8.705 | 7.467 | 1.733 | 0.623 |
| Opn4 KO | 10.000 | 350.000 | 8.800 | 2.700 | 3.367 | -0.110 |

| Figure 3 | |  |  |  |  |  |
| --- | --- | --- | --- | --- | --- | --- |
| Genotype | Sample Lux | Test Lux | Sample | Test (New) | Test (Old) | Ratio |
| C3H WT | 350.000 | 10.000 | 22.830 | 29.000 | 0.550 | 0.963 |
| C3H WT | 350.000 | 10.000 | 17.750 | 9.200 | 6.800 | 0.150 |
| C3H WT | 350.000 | 10.000 | 22.440 | 9.850 | 0.350 | 0.931 |
| C3H WT | 350.000 | 10.000 | 17.240 | 22.550 | 1.100 | 0.907 |
| C3H WT | 350.000 | 10.000 | 13.650 | 12.200 | 0.000 | 1.000 |
| Opn4 WT | 350.000 | 10.000 | 4.600 | 7.100 | 2.900 | 0.420 |
| Opn4 WT | 350.000 | 10.000 | 14.430 | 5.600 | 2.750 | 0.341 |
| Opn4 WT | 350.000 | 10.000 | 6.250 | 0.400 | 0.350 | 0.067 |
| Opn4 WT | 350.000 | 10.000 | 14.810 | 9.550 | 1.500 | 0.729 |
| Opn4 WT | 350.000 | 10.000 | 2.810 | 10.300 | 6.050 | 0.260 |
| C3H WT | 350.000 | 350.000 | 14.320 | 9.000 | 4.050 | 0.379 |
| C3H WT | 350.000 | 350.000 | 17.620 | 0.000 | 27.450 | -1.000 |
| C3H WT | 350.000 | 350.000 | 6.910 | 1.050 | 3.500 | -0.538 |
| C3H WT | 350.000 | 350.000 | 6.730 | 2.500 | 2.300 | 0.042 |
| C3H WT | 350.000 | 350.000 | 21.790 | 32.350 | 4.850 | 0.739 |
| Opn4 WT | 350.000 | 350.000 | 6.190 | 1.000 | 1.950 | -0.322 |
| Opn4 WT | 350.000 | 350.000 | 6.510 | 7.450 | 0.700 | 0.828 |
| Opn4 WT | 350.000 | 350.000 | 16.340 | 3.200 | 1.650 | 0.320 |
| Opn4 WT | 350.000 | 350.000 | 17.440 | 8.500 | 7.300 | 0.076 |
| Opn4 WT | 350.000 | 350.000 | 13.820 | 2.800 | 2.450 | 0.067 |
| C3H WT | 350.000 | 350.000 | 3.100 | 0.000 | 0.000 | 0.000 |
| C3H WT | 350.000 | 350.000 | 9.460 | 0.000 | 0.000 | 0.000 |
| C3H WT | 350.000 | 350.000 | 19.300 | 3.950 | 1.450 | 0.463 |
| C3H WT | 350.000 | 350.000 | 18.480 | 9.950 | 0.000 | 1.000 |
| C3H WT | 350.000 | 350.000 | 12.440 | 0.000 | 0.000 | 0.000 |
| Opn4 WT | 350.000 | 350.000 | 4.220 | 0.350 | 3.050 | -0.794 |
| Opn4 WT | 350.000 | 350.000 | 7.970 | 13.600 | 2.450 | 0.695 |
| Opn4 WT | 350.000 | 350.000 | 1.620 | 0.000 | 0.200 | -1.000 |
| Opn4 WT | 350.000 | 350.000 | 1.570 | 0.600 | 0.000 | 1.000 |
| rd/rd cl | 350.000 | 10.000 | 9.580 | 12.650 | 6.900 | 0.294 |
| rd/rd cl | 350.000 | 10.000 | 19.430 | 14.300 | 1.500 | 0.810 |
| rd/rd cl | 350.000 | 10.000 | 17.820 | 9.100 | 5.250 | 0.268 |
| rd/rd cl | 350.000 | 10.000 | 17.070 | 11.550 | 7.200 | 0.232 |
| rd/rd cl | 350.000 | 10.000 | 17.170 | 16.800 | 1.000 | 0.888 |
| rd/rd cl | 350.000 | 350.000 | 4.430 | 14.150 | 0.600 | 0.919 |
| rd/rd cl | 350.000 | 350.000 | 15.790 | 14.950 | 1.000 | 0.875 |
| rd/rd cl | 350.000 | 350.000 | 22.060 | 10.350 | 2.700 | 0.586 |
| rd/rd cl | 350.000 | 350.000 | 10.710 | 5.750 | 4.900 | 0.080 |
| rd/rd cl | 350.000 | 350.000 | 30.000 | 9.200 | 4.500 | 0.343 |
| rd/rd cl | 350.000 | 350.000 | 13.790 | 4.550 | 4.550 | 0.000 |
| rd/rd cl | 350.000 | 350.000 | 18.380 | 4.700 | 8.100 | -0.266 |
| rd/rd cl | 350.000 | 350.000 | 8.530 | 15.100 | 1.300 | 0.841 |
| rd/rd cl | 350.000 | 350.000 | 16.420 | 9.100 | 0.250 | 0.947 |
| rd/rd cl | 350.000 | 350.000 | 17.970 | 14.000 | 5.850 | 0.411 |
| Opn4 KO | 350.000 | 10.000 | 13.160 | 1.250 | 3.650 | -0.490 |
| Opn4 KO | 350.000 | 10.000 | 11.970 | 1.600 | 1.350 | 0.085 |
| Opn4 KO | 350.000 | 10.000 | 11.320 | 17.100 | 0.300 | 0.966 |
| Opn4 KO | 350.000 | 10.000 | 11.710 | 9.900 | 3.350 | 0.494 |
| Opn4 KO | 350.000 | 10.000 | 4.650 | 10.650 | 0.050 | 0.991 |
| Opn4 KO | 350.000 | 350.000 | 12.770 | 14.000 | 2.100 | 0.739 |
| Opn4 KO | 350.000 | 350.000 | 7.640 | 5.550 | 0.100 | 0.965 |
| Opn4 KO | 350.000 | 350.000 | 6.610 | 2.800 | 0.000 | 1.000 |
| Opn4 KO | 350.000 | 350.000 | 9.380 | 8.800 | 0.500 | 0.892 |
| Opn4 KO | 350.000 | 350.000 | 12.220 | 15.300 | 4.100 | 0.577 |
| Opn4 KO | 350.000 | 350.000 | 16.070 | 11.400 | 4.650 | 0.421 |
| Opn4 KO | 350.000 | 350.000 | 11.420 | 10.750 | 8.700 | 0.105 |
| Opn4 KO | 350.000 | 350.000 | 8.670 | 5.850 | 14.000 | -0.411 |
| Opn4 KO | 350.000 | 350.000 | 10.660 | 2.800 | 2.200 | 0.120 |
| Opn4 KO | 350.000 | 350.000 | 16.980 | 6.750 | 3.450 | 0.324 |

| Figure S2 | | | | |  |  |  |  |
| --- | --- | --- | --- | --- | --- | --- | --- | --- |
| Genotype | | Sample | Test (New) | | Test (Old) | Ratio |  |  |
| C3H WT | | 12.483 | 8.400 | | 18.233 | -0.369 |  |  |
| C3H WT | | 7.644 | 21.067 | | 2.433 | 0.793 |  |  |
| C3H WT | | 16.961 | 17.367 | | 12.433 | 0.166 |  |  |
| C3H WT | | 15.050 | 25.267 | | 10.733 | 0.404 |  |  |
| C3H WT | | 9.522 | 11.100 | | 2.033 | 0.690 |  |  |
| C3H WT | | 14.567 | 11.200 | | 2.033 | 0.693 |  |  |
| C3H WT | | 11.422 | 12.867 | | 6.367 | 0.338 |  |  |
| C3H WT | | 5.856 | 4.200 | | 0.767 | 0.691 |  |  |
| C3H WT | | 5.405 | 9.000 | | 0.733 | 0.849 |  |  |
| C3H WT | | 6.970 | 3.467 | | 0.000 | 1.000 |  |  |
| C3H WT | | 7.000 | 3.867 | | 1.567 | 0.423 |  |  |
| C3H WT | | 0.835 | 2.067 | | 0.000 | 1.000 |  |  |
| rd/rd cl | | 10.656 | 8.167 | | 15.367 | -0.306 |  |  |
| rd/rd cl | | 11.156 | 8.633 | | 10.833 | -0.113 |  |  |
| rd/rd cl | | 16.044 | 15.700 | | 16.133 | -0.014 |  |  |
| rd/rd cl | | 13.844 | 15.700 | | 6.967 | 0.385 |  |  |
| rd/rd cl | | 8.689 | 14.100 | | 6.900 | 0.343 |  |  |
| rd/rd cl | | 11.311 | 5.333 | | 4.333 | 0.103 |  |  |
| rd/rd cl | | 10.439 | 7.000 | | 9.367 | -0.145 |  |  |
| rd/rd cl | | 10.328 | 15.633 | | 8.333 | 0.305 |  |  |
| rd/rd cl | | 5.290 | 4.667 | | 2.933 | 0.228 |  |  |
| rd/rd cl | | 6.645 | 6.900 | | 10.767 | -0.219 |  |  |
| rd/rd cl | | 8.055 | 7.367 | | 22.967 | -0.514 |  |  |
| rd/rd cl | | 6.170 | 8.000 | | 2.633 | 0.505 |  |  |
| Opn4 WT | | 5.825 | 3.333 | | 0.933 | 0.563 |  |  |
| Opn4 WT | | 4.760 | 8.467 | | 0.533 | 0.881 |  |  |
| Opn4 WT | | 7.230 | 0.800 | | 0.000 | 1.000 |  |  |
| Opn4 WT | | 8.945 | 3.067 | | 0.000 | 1.000 |  |  |
| Opn4 WT | | 8.295 | 2.567 | | 1.967 | 0.132 |  |  |
| Opn4 WT | | 8.525 | 4.867 | | 1.567 | 0.513 |  |  |
| Opn4 WT | | 5.025 | 23.500 | | 1.933 | 0.848 |  |  |
| Opn4 WT | | 3.840 | 1.767 | | 3.667 | -0.350 |  |  |
| Opn4 KO | | 10.217 | 11.850 | | 1.750 | 0.743 |  |  |
| Opn4 KO | | 10.767 | 5.600 | | 1.950 | 0.483 |  |  |
| Opn4 KO | | 14.164 | 8.900 | | 11.750 | -0.138 |  |  |
| Opn4 KO | | 9.636 | 12.200 | | 7.100 | 0.264 |  |  |
| Opn4 KO | | 10.684 | 17.650 | | 1.100 | 0.883 |  |  |
| Opn4 KO | | 9.867 | 17.300 | | 13.450 | 0.125 |  |  |
| Opn4 KO | | 12.483 | 6.800 | | 1.400 | 0.659 |  |  |
| Opn4 KO | | 10.394 | 4.350 | | 1.400 | 0.513 |  |  |
|  | |  |  | |  |  |  |  |
| Genotype | | 0.1 c/d | 0.3 c/d | | 0.4 c/d |  |  |  |
| C3H WT | | 4.000 | 5.000 | | 1.000 |  |  |  |
| C3H WT | | 2.000 | 4.000 | | 2.000 |  |  |  |
| C3H WT | | 6.000 | 7.000 | | 3.000 |  |  |  |
| C3H WT | | 4.000 | 5.000 | | 1.000 |  |  |  |
| rd/rd cl | | 0.000 | 0.000 | | 0.000 |  |  |  |
| rd/rd cl | | 1.000 | 0.000 | | 0.000 |  |  |  |
| rd/rd cl | | 0.000 | 1.000 | | 0.000 |  |  |  |
| rd/rd cl | | 0.000 | 1.000 | | 0.000 |  |  |  |
| Opn4 WT | | 9.000 | 5.000 | | 5.000 |  |  |  |
| Opn4 WT | | 6.000 | 8.000 | | 4.000 |  |  |  |
| Opn4 WT | | 8.000 | 4.000 | | 5.000 |  |  |  |
| Opn4 WT | | 10.000 | 5.000 | | 6.000 |  |  |  |
| Opn4 KO | | 7.000 | 6.000 | | 5.000 |  |  |  |
| Opn4 KO | | 7.000 | 7.000 | | 3.000 |  |  |  |
| Opn4 KO | | 5.000 | 6.000 | | 5.000 |  |  |  |
| Opn4 KO | | 8.000 | 6.000 | | 8.000 |  |  |  |
|  | |  |  | |  |  |  |  |
| Figure S3 | | | | |  |  |  |  |
| Strain | Sample Lux | | | Test Lux | Sample | Test (New) | Test (Old) | Ratio |
| C3H WT | 10.000 | | | 10.000 | 10.920 | 11.100 | 0.050 | 0.991 |
| C3H WT | 10.000 | | | 10.000 | 10.825 | 7.067 | 2.367 | 0.498 |
| C3H WT | 10.000 | | | 10.000 | 5.550 | 8.333 | 0.000 | 1.000 |
| C3H WT | 10.000 | | | 10.000 | 5.195 | 15.200 | 0.000 | 1.000 |
| C3H WT | 10.000 | | | 10.000 | 4.345 | 2.000 | 0.033 | 0.967 |
| C3H WT | 10.000 | | | 10.000 | 3.630 | 11.800 | 0.000 | 1.000 |
| Opn4 WT | 10.000 | | | 10.000 | 6.135 | 5.900 | 1.100 | 0.686 |
| Opn4 WT | 10.000 | | | 10.000 | 14.735 | 8.400 | 15.500 | -0.297 |
| Opn4 WT | 10.000 | | | 10.000 | 8.610 | 23.500 | 8.700 | 0.460 |
| Opn4 WT | 10.000 | | | 10.000 | 9.605 | 5.800 | 0.300 | 0.902 |
| Opn4 WT | 10.000 | | | 10.000 | 3.255 | 1.333 | 0.167 | 0.778 |
| Opn4 WT | 10.000 | | | 10.000 | 4.405 | 3.967 | 0.033 | 0.983 |
| C3H WT | 10.000 | | | 10.000 | 12.890 | 9.250 | 0.000 | 1.000 |
| C3H WT | 10.000 | | | 10.000 | 9.850 | 6.450 | 4.800 | 0.147 |
| C3H WT | 10.000 | | | 10.000 | 7.320 | 6.150 | 0.100 | 0.968 |
| C3H WT | 10.000 | | | 10.000 | 12.860 | 47.450 | 0.000 | 1.000 |
| C3H WT | 10.000 | | | 10.000 | 17.290 | 26.350 | 0.000 | 1.000 |
| Opn4 WT | 10.000 | | | 10.000 | 3.940 | 4.950 | 0.150 | 0.941 |
| Opn4 WT | 10.000 | | | 10.000 | 13.050 | 14.750 | 6.400 | 0.395 |
| Opn4 WT | 10.000 | | | 10.000 | 7.280 | 3.900 | 0.950 | 0.608 |
| Opn4 WT | 10.000 | | | 10.000 | 5.510 | 12.550 | 2.150 | 0.707 |
| Opn4 WT | 10.000 | | | 10.000 | 10.780 | 7.600 | 6.650 | 0.067 |
| C3H WT | 350.000 | | | 10.000 | 22.830 | 29.000 | 0.550 | 0.963 |
| C3H WT | 350.000 | | | 10.000 | 17.750 | 9.200 | 6.800 | 0.150 |
| C3H WT | 350.000 | | | 10.000 | 22.440 | 9.850 | 0.350 | 0.931 |
| C3H WT | 350.000 | | | 10.000 | 17.240 | 22.550 | 1.100 | 0.907 |
| C3H WT | 350.000 | | | 10.000 | 13.650 | 12.200 | 0.000 | 1.000 |
| Opn4 WT | 350.000 | | | 10.000 | 4.600 | 7.100 | 2.900 | 0.420 |
| Opn4 WT | 350.000 | | | 10.000 | 14.430 | 5.600 | 2.750 | 0.341 |
| Opn4 WT | 350.000 | | | 10.000 | 6.250 | 0.400 | 0.350 | 0.067 |
| Opn4 WT | 350.000 | | | 10.000 | 14.810 | 9.550 | 1.500 | 0.729 |
| Opn4 WT | 350.000 | | | 10.000 | 2.810 | 10.300 | 6.050 | 0.260 |
| C3H WT | 10.000 | | | 350.000 | 11.170 | 7.667 | 2.733 | 0.474 |
| C3H WT | 10.000 | | | 350.000 | 12.280 | 2.400 | 2.667 | -0.053 |
| C3H WT | 10.000 | | | 350.000 | 3.570 | 4.600 | 0.000 | 1.000 |
| C3H WT | 10.000 | | | 350.000 | 6.405 | 14.633 | 0.000 | 1.000 |
| C3H WT | 10.000 | | | 350.000 | 6.910 | 0.000 | 54.733 | -1.000 |
| C3H WT | 10.000 | | | 350.000 | 4.465 | 0.000 | 19.867 | -1.000 |
| Opn4 WT | 10.000 | | | 350.000 | 10.530 | 0.600 | 4.100 | -0.745 |
| Opn4 WT | 10.000 | | | 350.000 | 11.990 | 14.900 | 5.500 | 0.461 |
| Opn4 WT | 10.000 | | | 350.000 | 6.400 | 1.600 | 10.100 | -0.726 |
| Opn4 WT | 10.000 | | | 350.000 | 5.550 | 17.600 | 4.900 | 0.564 |
| Opn4 WT | 10.000 | | | 350.000 | 6.915 | 3.100 | 10.700 | -0.551 |
| Opn4 WT | 10.000 | | | 350.000 | 2.790 | 2.000 | 1.100 | 0.290 |
| C3H WT | 350.000 | | | 350.000 | 14.320 | 9.000 | 4.050 | 0.379 |
| C3H WT | 350.000 | | | 350.000 | 17.620 | 0.000 | 27.450 | -1.000 |
| C3H WT | 350.000 | | | 350.000 | 6.910 | 1.050 | 3.500 | -0.538 |
| C3H WT | 350.000 | | | 350.000 | 6.730 | 2.500 | 2.300 | 0.042 |
| C3H WT | 350.000 | | | 350.000 | 21.790 | 32.350 | 4.850 | 0.739 |
| Opn4 WT | 350.000 | | | 350.000 | 6.190 | 1.000 | 1.950 | -0.322 |
| Opn4 WT | 350.000 | | | 350.000 | 6.510 | 7.450 | 0.700 | 0.828 |
| Opn4 WT | 350.000 | | | 350.000 | 16.340 | 3.200 | 1.650 | 0.320 |
| Opn4 WT | 350.000 | | | 350.000 | 17.440 | 8.500 | 7.300 | 0.076 |
| Opn4 WT | 350.000 | | | 350.000 | 13.820 | 2.800 | 2.450 | 0.067 |
| C3H WT | 350.000 | | | 350.000 | 3.100 | 0.000 | 0.000 | 0.000 |
| C3H WT | 350.000 | | | 350.000 | 9.460 | 0.000 | 0.000 | 0.000 |
| C3H WT | 350.000 | | | 350.000 | 19.300 | 3.950 | 1.450 | 0.463 |
| C3H WT | 350.000 | | | 350.000 | 18.480 | 9.950 | 0.000 | 1.000 |
| C3H WT | 350.000 | | | 350.000 | 12.440 | 0.000 | 0.000 | 0.000 |
| Opn4 WT | 350.000 | | | 350.000 | 4.220 | 0.350 | 3.050 | -0.794 |
| Opn4 WT | 350.000 | | | 350.000 | 7.970 | 13.600 | 2.450 | 0.695 |
| Opn4 WT | 350.000 | | | 350.000 | 1.620 | 0.000 | 0.200 | -1.000 |
| Opn4 WT | 350.000 | | | 350.000 | 1.570 | 0.600 | 0.000 | 1.000 |

| Figure S4 | | |  |  |  |
| --- | --- | --- | --- | --- | --- |
| Genotype | Lux | Closed Time | Open Time | Closed Entry | Open Entry |
| C3H WT | 350.000 | 0.960 | 0.040 | 0.625 | 0.375 |
| C3H WT | 350.000 | 0.998 | 0.002 | 0.923 | 0.077 |
| C3H WT | 350.000 | 0.827 | 0.173 | 0.522 | 0.478 |
| C3H WT | 350.000 | 0.839 | 0.161 | 0.657 | 0.343 |
| C3H WT | 350.000 | 0.350 | 0.650 | 0.389 | 0.611 |
| C3H WT | 350.000 | 0.738 | 0.262 | 0.630 | 0.370 |
| C3H WT | 350.000 | 0.898 | 0.102 | 0.667 | 0.333 |
| C3H WT | 350.000 | 0.883 | 0.117 | 0.519 | 0.481 |
| C3H WT | 10.000 | 0.956 | 0.044 | 0.766 | 0.234 |
| C3H WT | 10.000 | 0.886 | 0.114 | 0.761 | 0.239 |
| C3H WT | 10.000 | 0.181 | 0.819 | 0.474 | 0.526 |
| C3H WT | 10.000 | 0.786 | 0.214 | 0.593 | 0.407 |
| C3H WT | 10.000 | 1.000 | 0.000 | 1.000 | 0.000 |
| C3H WT | 10.000 | 0.956 | 0.044 | 0.774 | 0.226 |
| C3H WT | 10.000 | 0.994 | 0.006 | 0.944 | 0.056 |
| C3H WT | 10.000 | 0.882 | 0.118 | 0.745 | 0.255 |
| rd/rd cl | 350.000 | 0.890 | 0.110 | 0.600 | 0.400 |
| rd/rd cl | 350.000 | 0.465 | 0.535 | 0.339 | 0.661 |
| rd/rd cl | 350.000 | 0.736 | 0.264 | 0.632 | 0.368 |
| rd/rd cl | 350.000 | 0.430 | 0.570 | 0.346 | 0.654 |
| rd/rd cl | 350.000 | 0.000 | 1.000 | 0.000 | 1.000 |
| rd/rd cl | 10.000 | 0.260 | 0.740 | 0.425 | 0.575 |
| rd/rd cl | 10.000 | 0.376 | 0.624 | 0.357 | 0.643 |
| rd/rd cl | 10.000 | 0.401 | 0.599 | 0.466 | 0.534 |
| rd/rd cl | 10.000 | 0.565 | 0.435 | 0.492 | 0.508 |
| rd/rd cl | 10.000 | 0.065 | 0.935 | 0.232 | 0.768 |
| Opn4 WT | 350.000 | 0.623 | 0.377 | 0.519 | 0.481 |
| Opn4 WT | 350.000 | 0.749 | 0.251 | 0.607 | 0.393 |
| Opn4 WT | 350.000 | 0.623 | 0.377 | 0.459 | 0.541 |
| Opn4 WT | 350.000 | 0.700 | 0.300 | 0.528 | 0.472 |
| Opn4 WT | 350.000 | 0.849 | 0.151 | 0.600 | 0.400 |
| Opn4 WT | 350.000 | 0.475 | 0.525 | 0.500 | 0.500 |
| Opn4 WT | 10.000 | 0.858 | 0.142 | 0.714 | 0.286 |
| Opn4 WT | 10.000 | 0.824 | 0.176 | 0.579 | 0.421 |
| Opn4 WT | 10.000 | 0.849 | 0.151 | 0.625 | 0.375 |
| Opn4 WT | 10.000 | 0.382 | 0.618 | 0.436 | 0.564 |
| Opn4 WT | 10.000 | 0.677 | 0.323 | 0.500 | 0.500 |
| Opn4 KO | 350.000 | 0.620 | 0.380 | 0.559 | 0.441 |
| Opn4 KO | 350.000 | 0.376 | 0.624 | 0.413 | 0.587 |
| Opn4 KO | 350.000 | 0.623 | 0.377 | 0.391 | 0.609 |
| Opn4 KO | 350.000 | 0.839 | 0.161 | 0.533 | 0.467 |
| Opn4 KO | 350.000 | 0.721 | 0.279 | 0.538 | 0.462 |
| Opn4 KO | 10.000 | 0.518 | 0.482 | 0.472 | 0.528 |
| Opn4 KO | 10.000 | 0.949 | 0.051 | 0.759 | 0.241 |
| Opn4 KO | 10.000 | 0.670 | 0.330 | 0.571 | 0.429 |
| Opn4 KO | 10.000 | 0.749 | 0.251 | 0.484 | 0.516 |

| Figure S5 | | |  |  |  |
| --- | --- | --- | --- | --- | --- |
| Genotype | Lux | Dark Time | Bright Time | Dark Entry | Bright Entry |
| Opn4 WT | 10.000 | 0.577 | 0.423 | 0.529 | 0.471 |
| Opn4 WT | 10.000 | 0.744 | 0.256 | 0.520 | 0.480 |
| Opn4 WT | 10.000 | 0.747 | 0.253 | 0.545 | 0.455 |
| Opn4 WT | 10.000 | 0.851 | 0.149 | 0.533 | 0.467 |
| Opn4 WT | 350.000 | 0.883 | 0.117 | 0.556 | 0.444 |
| Opn4 WT | 350.000 | 0.960 | 0.040 | 1.000 | 0.000 |
| Opn4 WT | 350.000 | 0.796 | 0.204 | 0.538 | 0.462 |
| Opn4 WT | 350.000 | 0.776 | 0.224 | 0.500 | 0.500 |
| Opn4 WT | 350.000 | 0.898 | 0.102 | 0.667 | 0.333 |
| C3H WT | 10.000 | 0.213 | 0.787 | 0.571 | 0.429 |
| C3H WT | 10.000 | 0.712 | 0.288 | 0.600 | 0.400 |
| C3H WT | 10.000 | 0.927 | 0.073 | 1.000 | 0.000 |
| C3H WT | 10.000 | 0.001 | 0.999 | 1.000 | 0.000 |
| C3H WT | 10.000 | 0.249 | 0.751 | 0.571 | 0.429 |
| C3H WT | 350.000 | 0.866 | 0.134 | 1.000 | 0.000 |
| C3H WT | 350.000 | 0.001 | 0.999 | 1.000 | 0.000 |
| C3H WT | 350.000 | 0.747 | 0.253 | 0.667 | 0.333 |
| C3H WT | 350.000 | 0.810 | 0.190 | 0.600 | 0.400 |
| C3H WT | 350.000 | 0.705 | 0.295 | 0.571 | 0.429 |
| rd/rd cl | 10.000 | 0.358 | 0.642 | 0.526 | 0.474 |
| rd/rd cl | 10.000 | 0.266 | 0.734 | 0.526 | 0.474 |
| rd/rd cl | 10.000 | 0.481 | 0.519 | 0.529 | 0.471 |
| rd/rd cl | 10.000 | 0.000 | 1.000 | 0.500 | 0.500 |
| rd/rd cl | 10.000 | 0.310 | 0.690 | 0.500 | 0.500 |
| rd/rd cl | 350.000 | 0.381 | 0.619 | 0.500 | 0.500 |
| rd/rd cl | 350.000 | 0.361 | 0.639 | 0.533 | 0.467 |
| rd/rd cl | 350.000 | 0.542 | 0.458 | 0.500 | 0.500 |
| rd/rd cl | 350.000 | 0.393 | 0.607 | 0.500 | 0.500 |
| rd/rd cl | 350.000 | 0.648 | 0.352 | 0.500 | 0.500 |
| Opn4 KO | 10.000 | 0.792 | 0.208 | 0.533 | 0.467 |
| Opn4 KO | 10.000 | 0.705 | 0.295 | 0.545 | 0.455 |
| Opn4 KO | 10.000 | 0.390 | 0.610 | 0.524 | 0.476 |
| Opn4 KO | 10.000 | 0.760 | 0.240 | 0.538 | 0.462 |
| Opn4 KO | 10.000 | 0.612 | 0.388 | 0.529 | 0.471 |
| Opn4 KO | 350.000 | 0.808 | 0.192 | 0.500 | 0.500 |
| Opn4 KO | 350.000 | 0.741 | 0.259 | 0.538 | 0.462 |
| Opn4 KO | 350.000 | 0.607 | 0.393 | 0.556 | 0.444 |
| Opn4 KO | 350.000 | 0.827 | 0.173 | 0.556 | 0.444 |
| Opn4 KO | 350.000 | 0.262 | 0.738 | 0.538 | 0.462 |
